# A three-player game model for promoting enterprise green technology innovation from the perspective of media coverage

**DOI:** 10.3389/fpubh.2023.1253247

**Published:** 2024-02-08

**Authors:** Yan Liu, Yun-ping Chen, Tong-ping Xie, Yi-han Xia

**Affiliations:** ^1^School of Economics and Management, Jingdezhen University, Jingdezhen, China; ^2^Management Science and Engineering Research Center, Jiangxi Normal University, Nanchang, China; ^3^School of Economics and Management, Gongqing Institute of Science and Technology, Gongqing, China

**Keywords:** media coverage, government regulation, public supervision, green technology innovation, evolutionary game

## Abstract

**Objective:**

The objective of this study was to explore the game relationship among enterprise, the government, and the public under the new media environment, so as to provide decision-making reference for improving enterprise green technology innovation and promoting economy high-quality development with new media participation.

**Methods:**

This study constructs a three-subject evolutionary game model of enterprise, government, and public based on multi-agent relationship analysis and evolutionary game theory. In addition, the derivation of an evolutionary equilibrium strategy and numerical simulation analysis is carried out to comprehensively explore the evolution trajectory of green technology innovation system under the new media environment.

**Findings:**

(1) The system may have four stable evolutionary strategies: (1,0,0), (0,0,1), (1,0,1), and (1,1,1). (2) The initial strategy probability of various actors would affect the system evolution speed but not the evolution result, and the authenticity of new media reports is an important factor determining the system evolution of green technology innovation. (3) Numerical simulation results show that a fair and just new media environment can effectively constrain the traditional production behavior of enterprise, actively guide the public to participate in supervision, and play an alternative role to government regulation to a certain extent.

**Value:**

This study explores the evolutionary balance strategy of green technology innovation system under the new media environment, which not only enriches relevant theories of media environment governance but also has important reference value for promoting enterprises’ green technology innovation and establishing an environmental governance system jointly governed by government, enterprise, public, and media.

## 1 Introduction

As the core body to promote the development of the national economy, enterprises generate high GDP growth while their extensive production modes have many negative impacts on the ecological environment, prompting the government to prioritize technological innovation and environmental protection. To guide the market-oriented development of green technology innovation, the National Development and Reform Commission and the Ministry of Science and Technology jointly issued the “Guiding Opinions on Building a Market-oriented Green Technology Innovation System” in 2019. The International Energy Agency (IEA) also stated that green technology is expected to play a key role in reducing carbon emissions and mitigating climate change. Green technology innovation not only has economic characteristics such as improving production efficiency and enterprise competitiveness but also has social characteristics such as environmental protection and emission reduction, making it the key to resolving the conflict between national economic growth and ecological governance.

Due to the inherent defects of green technology innovation such as high investment cost, long cycle, high risk, and double externalities, enterprises are generally difficult to form spontaneously and need to rely on external forces to promote it (1). Porter’s hypothesis holds that appropriate environmental regulations can promote enterprises’ green technology innovation (2), and the effectiveness of environmental regulations on enterprises’ green technology innovation has been confirmed by many scholars (3, 4). With the improvement of information technology and the development of social media, the influence of media opinion on enterprises’ green production and environmental governance has also aroused widespread discussion. Chen et al. (5) pointed out that the transparency of information brought by media attention and the pressure of public opinion have a direct effect on pollution control of enterprises and thus promote green innovation of enterprises. Harness (6) believe that enterprises with environmental accidents are more likely to attract media attention, which will lead to consumer boycott, social condemnation, and government punishment. Media coverage has become an important factor that cannot be ignored to promote enterprise green technology innovation. However, there are few studies on the influence and mechanism of media coverage on enterprises’ green technology innovation. Li et al. (7) pointed out by building a reputation community framework that the pressure of public opinion generated by media coverage not only affects the enterprise itself but also has an impact on the government, the public, and other relevant groups. Based on stakeholder theory, Tang et al. (8) confirmed that media coverage will affect enterprises’ green technology innovation strategies by regulating government and public behaviors. Can media coverage influence a firm’s behavior strategy? How does media coverage influence government regulation and public participation to promote corporate green technology innovation? In view of the above problems, from the perspective of media attention, this study discusses the game relationship between enterprises, government, and the public based on stakeholder analysis and evolutionary game theory, so as to provide a reference for effectively promoting enterprises’ green technology innovation and establishing an environmental governance system jointly governed by government, enterprises, public, and media.

## 2 Literature review

Green technological innovation generally refers to the technological innovation behavior carried out in accordance with the law of ecological economic development for the purpose of reducing or eliminating ecological environmental pollution, saving resources and energy, and protecting the environment (9). As an ecosystem engineering with multiple subjects, green technology innovation has a complex strategy evolution that has recently been studied by scholars both at home and abroad. The early studies were based on the two-subject game between the government and enterprise. Wang et al. (10), Nie et al. (11), and Xu et al. (12) concluded by developing a game model between the government and enterprise that whether the enterprise carries out green technology innovation is determined by the benefits and costs of the game between the government and enterprise. The incremental costs of green technology innovation, the government’s reward and punishment regulation for enterprise, and the costs of government supervision will all have an impact on the system’s evolution results. With an increasing number of negative externalities in the enterprise’s operation process, the defects of the government’s “unified” regulation mode have become emerged, such as high supervision costs, a limited number of technicians, and law enforcement personnel. Green technology innovation behavior frequently involves multiple subjects such as the government, the public, and enterprise, and researchers may draw biased conclusions if they only focus on the driving effect of government behavior (13). The academic community has focused on the problem of enterprise green technology innovation with multi-agent, particularly the tripartite game among the government, enterprise, and the public. The public has both market-oriented and behavioral supervision effects on enterprise behavior, according to Li et al. (14) and Xu et al. (15), and both public coverage and public reporting may have a driving effect on green technology innovation. Cao et al. (16) and Encarnacao et al. (17) constructed a three-subject evolutionary game model involving the government, enterprise, and the public to investigate the public’s role mechanism in green technology innovation. Qu et al. (18) and Guan et al. (19) studied the evolution strategies of the government, the public, and the enterprise and discovered that they are not only related to the initial participation ratio of each subject but also affected by the value of their related parameters. A small number of scholars have found that the behaviors of green organizations, scientific research institutions, and financial institutions will also have an impact on the enterprise’s green technology innovation strategy (20–22).

There are abundant research studies on the tripartite game between the government, enterprises, and the public to promote green technology innovation. However, with the continuous update of information technology, the way and direction of public opinion communication have undergone great changes, and the influence of public opinion pressure reported by the media on green technology innovation cannot be underestimated. Environmental governance is a comprehensive action involving many interest groups such as the government, enterprises, the public, and the media (23). Ling et al. (24) and Zhang et al. (25) pointed out that as an important extra-legal system and an important social supervision subject in the construction of ecological civilization, the media, with its advantages of timely dissemination, wide coverage, and strong public opinion effect, can form a good interactive mechanism with government regulation, market regulation, and public participation. Ji et al. (26) and Xin (27) found that positive media reports can stimulate enterprises to disclose environmental information to promote green production, and media criticism and questioning will cause reputational pressure on enterprises and force them to reduce ecological pollution activities. Li et al. (28) believe that media play a role of value guidance and education supervision for the public in ecological environmental protection, and at the same time, the advantages of media can create a good public opinion atmosphere and social environment for ecological environmental protection. Li et al. (29) observed that media scrutiny can influence the strategy choices of enterprise, the public, and the government to some extent. Vaast et al. (30) pointed out that media coverage, as one of the important methods of external supervision, might reduce enterprise violations and help to enhance the effectiveness of government regulation. To sum up, it can be concluded that new media plays an important role in the process of green technology innovation of enterprises. Media coverage is not only an important way to scientifically guide and cultivate public awareness of green environmental protection but also an important way to restrict enterprises’ bad production behaviors through information disclosure and can also be used as a supplement to the regulatory functions of the government (31).

Although the above research has carried out a more comprehensive analysis of the game of green technology innovation under the new media environment, there are still the following deficiencies: First, the current research mainly focuses on the impact of government regulatory means on enterprises’ green innovation behavior, while less consideration is given to the role of the public in green technology innovation and the impact of the symbiotic conflict between the government and the public on enterprises’ behavior. Second, the existing research studies on the impact of new media on enterprises’ green technology innovation are mostly based on theoretical discussion or single quantitative research, and few are based on game theory to study the impact of the new media environment on the strategies of green technology innovation-related entities. Therefore, on the basis of existing studies, this study integrates the influence of the new media environment into the evolutionary game model of the government, enterprises, and the public, analyzes the evolutionary equilibrium strategy of the green technology innovation system under the new media environment, and further explores the influence of the initial probability value and the change of media report accuracy rate on the behavioral strategy and system evolution trajectory of the government, enterprises, and the public through numerical simulation.

The paper’s research structure is as follows: The second part is the discussion on the mechanism of action among relevant actors of green technology innovation; the third part is the construction of evolutionary game model (including the proposal of research hypotheses and the construction of three-party game model); the fourth part is the analysis of evolutionary equilibrium (including the analysis of evolutionary equilibrium of relevant actors’ strategies and the analysis of evolutionary equilibrium of green technology innovation system); the fifth part is the numerical simulation and finally the conclusion and enlightenment.

## 3 Analysis of the subject benefits

According to Agle et al. (32) definition of “*stakeholders*,” stakeholders of green technology innovation are all relevant individuals or groups who can influence the realization of green technology innovation goals. From the *genetic theory* (33), a change in one subject’s interest will result in a change in the occurrence mechanism of green technology innovation. Therefore, clarifying the interest relationship among the topics of green technology innovation is necessary to maximize the interests of all parties to support enterprise green technology innovation. In this research, green technology innovation under the new media environment involves the government, enterprise, the public, and media, and their interest demands and goals differ slightly.

Enterprise is the main producer of the market economy, and the motivation of green technology innovation can be divided into internal and external factors. The internal factors include maximization of economic benefits, enterprise social cognition, and corporate image, while the external factors include scientific and technological progress, policy driving force, market competition, and market demand (34). As a typical profit-oriented subject, enterprise seeks to pursue economic rewards while ignoring the ecological environment. For example, extensive production technology, illegal dumping of production wastes, and other behaviors of enterprise cause environmental pollution and waste of resources. Therefore, the government makes relevant policies, such as levying environmental taxes and fines to prevent enterprises from bad production behaviors and encouraging enterprise green technology innovation through innovation subsidies. At the same time, the public will actively supervise to find a balance between the amount of compensation for environmental pollution and the negative effects of pollution.

As the essential external environmental manager, the government’s regulation means will have an effect on the enterprise’s green innovation behavior. Pollution taxes for enterprise environmental pollution and R&D subsidies for green technology innovation will affect enterprise final profitability and thus change their behavior strategies. At the same time, the government will provide certain subsidies to support the public supervision. However, from the perspective of the government itself, regulation behavior by the government involves a large amount of manpower, material resources, and financial resources, will lower the company earnings, and hence affect the region’s economic development. The work efficiency of the government is directly linked to local economic development. Therefore, under the separation of powers system, the government faces an interest game between choosing no regulation for regional economic development and deciding to regulation based on the maximization of social welfare.

Economic progress cannot be separated from every member of the public, and the public demands drive consumption to form the economy. Therefore, the public’s green lifestyle and green consumption mode are the important pulling force for enterprise green technology innovation. At the same time, as the main beneficiaries of a good ecological environment and the direct victims of environmental pollution, the public has a supervisory responsibility over enterprise and government behavior. If the public participates in the supervision and reporting when enterprise adopts the traditional technology production, the public may get government subsidies, and the enterprise may compensate the public to maintain its social image. However, because public supervision needs to pay costs, the public behavior strategy is the interest game under the supervision costs and the supervision utilities.

As a “third party force,” new media are one of the key groups in promoting enterprise green technology innovation. The pressure of media opinions brought by both positive and negative reports can affect the efficiency of green technology innovation of enterprise through reputation orientation. The public relies on new media reports to understand environmental information, so media is an important role in guiding and cultivating public awareness of environmental protection. Simultaneously, the media will play the role of inspection and assistance in achieving good cooperation with the government to relieve the government’s regulatory pressure. However, as a for-profit organization, the media’s behavior strategy must assess the benefits and risks posed by the report. In addition, due to the restriction of supervision power, enterprise obstruction, and media personnel’s knowledge and ability, new media reports may have distortion phenomenon, which may mislead enterprise, government, and public behavior judgment.

**Figure 1:**
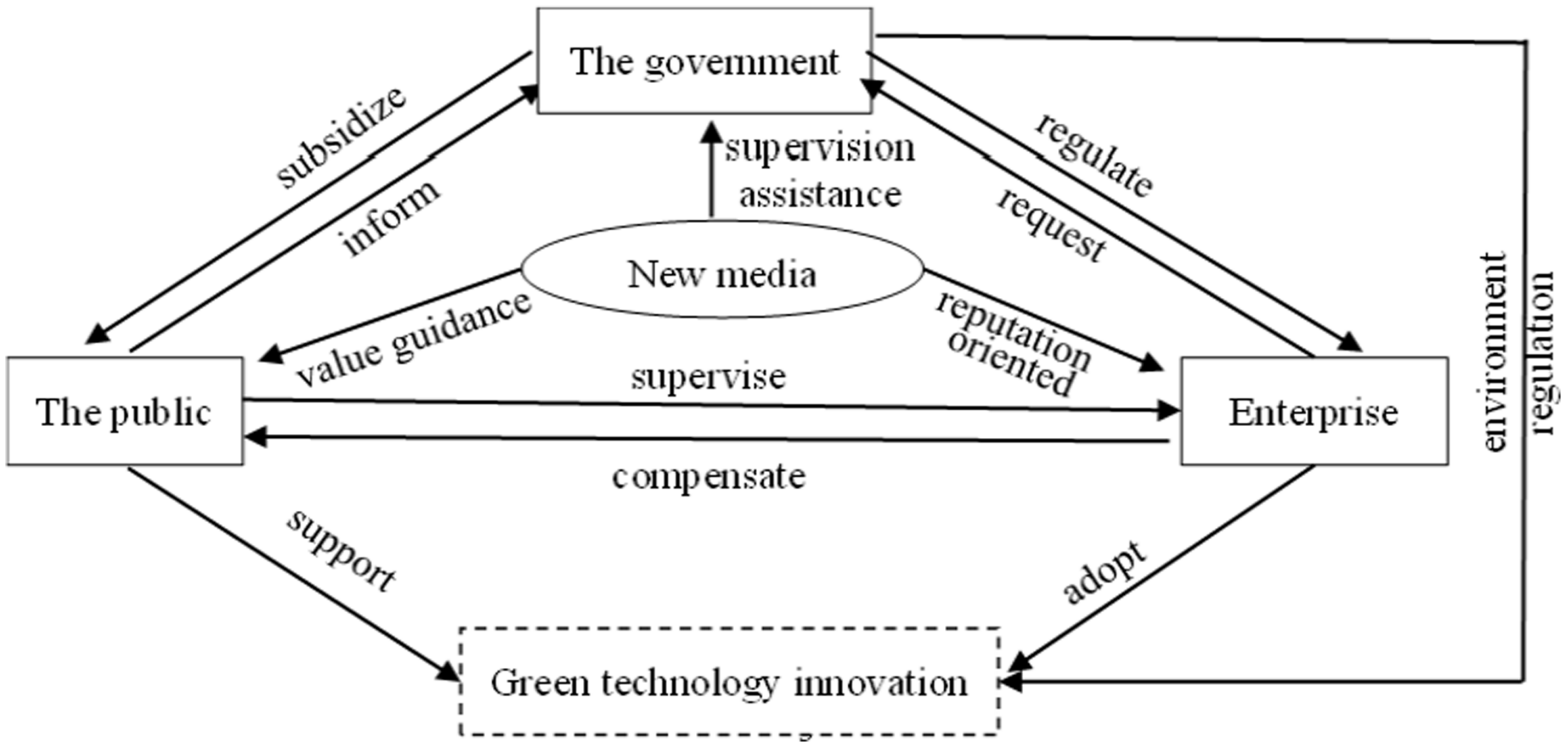
Relevant subjects and action mechanism of green technology innovation.

Thus, the theoretical system of the action mechanism among relevant subjects of green technology innovation can be obtained, as shown in Figure 1.

## 4 Evolutionary game analysis

### 4.1 Tripartite evolutionary game model formulation

#### 4.1.1 Research hypotheses

This research considers a green technology innovation system composed of enterprise, the government, and the public under the new media environment. All subjects have bounded rationality and will eventually reach a stable strategy through continuous learning and improvement. Based on this, the existing hypotheses are as follows:

**Hypothesis 1**: Enterprise, the government, or the public has two behavior strategies: Enterprise can choose to carry out green technology innovation or maintain traditional technology production. Assuming that the probability of adopting the green technology innovation strategy of the enterprise is *x*, then the probability of adopting traditional technology is 1-*x*; the government can choose to regulate or not regulate. Assuming that the probability of regulation for the government is *y*, the probability of no regulation is 1-*y*. The public can decide to participate in supervision or not, and assuming the probability of participating in supervision is *z*, the probability of not supervision is 1-*z*. Here, 0 ≤ {*x*, *y*, *z*} ≤1, and they are functions of time *t*.

**Hypothesis 2**: When the government chooses environmental regulation, it must pay the costs of regulation, which include R&D rewards *Cb* for enterprise green technology innovation and the subsidies *R_b_* for public supervision. Furthermore, the government can charge fines *C_s_* for traditional technology production of the enterprise. Generally speaking, the fines are higher than the R&D rewards and subsidies, namely, *Cs* > *Cb*, *Cs* > *Rb*; when the government decides not to regulate, its costs are zero.

**Hypothesis 3**: Enterprise can obtain general revenue *R0* from traditional technology production. It needs to pay additional incremental costs *C* for green technology innovation, but it may obtain incremental revenues *R*, in general, *R* > *C*. When an enterprise carries out green technology innovation, the government and the public gain from the environmental benefits *Re* and R*s;* however, they will suffer e*nvir*onmental losses *Be* and *Bs* when an enterprise chooses to produce with traditional technology.

**Hypothesis 4**: Public supervision needs to pay supervision costs *Cp*. Under the environment of public supervision, an enterprise choosing the green technology innovation can get reputation awards *Br*, and government regulation can obtain a good social reputation *Cr*; however, if enterprise carries on traditional technology production, it will suffer reputation losses *Bl* and need to pay environmental compensations *Ce* to the public, and the government will suffer reputational losses *Cl* if it neglects to regulate at this time.

**Hypothesis 5**: The information dissemination and opinion function of new media can raise public awareness of environmental protection, and the public who participates in supervision can receive green utilities *Rp*. However, media reports are just a new and effective channel for the public to play the supervisory role. Only when the public participates in supervision, can the new media environment play a role. While the public chooses not to supervise, the media cannot exert their influence on opinion. Given that new media has limited access to information about the subjects’ behavior, it is assumed that when the government decides to regulate, media can correctly identify enterprise behavior, and if the government decides no regulation, there will be distortion in new media reports (for example, false information reported by publics, deliberate smear by competitors, and false publicity by enterprises), and it could only correctly identify and report enterprise behavior with a probability of *f*. The parameter and variable symbols and their meanings are shown in Table 1 (35, 36).

**Table 1:** Parameters and variable symbol descriptions.

Parameters	Descriptions
*Cb*	R&D rewards for enterprise green technology innovation from government regulation
*Rb*	Fines charged for traditional technology of enterprise to government regulation
*Cs*	Green subsidies for public supervision from government regulation
*R0*	The general revenues obtained by the traditional technology production of enterprise
*R*	Incremental revenues from enterprise green technology innovation
*C*	Incremental costs of enterprise green technology innovation
*Re*	Environmental benefits obtained by the government when enterprise adopts green technology innovation
*Rs*	Environmental benefits obtained by the public when enterprise adopts green technology innovation
*Be*	The environmental losses brought by traditional technology production to the government
*Bs*	The environmental losses brought by traditional technology production to the public
*Cp*	Costs of public supervision
*Ce*	The compensations of enterprise traditional technology behavior to public supervision when the government chooses to regulate
*Cr*	The reputational rewards obtained by the government for effective regulation (enterprise green technology innovation) when the public chooses to supervise
*Cl*	The reputational damages to the government for no regulation (enterprises traditional technology production) when the public chooses to supervise
*Bl*	The reputational damages to the enterprise for traditional technology production when the public chooses to supervise
*Br*	The reputational rewards obtained by the government for enterprise green technology innovation when the public chooses to supervise
*Rp*	Green utilities obtained by public supervision when new media are involved
*f*	The correct rate of new media coverage

According to the research hypothesis and game theory, the payoff matrix of enterprise, the government, and the public under the new media environment can be obtained, as shown in Table 2.

**Table 2:** Payoff matrix among government–enterprise–public.

		**Enterprise**			
		Green technology innovation	Traditional technology production		
The government	Regulation	*R0 + R + Cb-C + Br;* *Cr + Re-Cb-Cs;* *Rp + Rs + Cs -Cp*	*R0-Rb-Ce-Bl;* *Rb-Cs-Be;* *Rp + Cs-Bs-Cp + Ce*	Supervision	The public
		*R0 + R + Cb-C;* *Re-Cb;* *Rs*	*R0-Rb;* *Rb-Be;* *-Bs*	No supervision	
	No regulation	R0 + R-C + f*Br-(1*-f*)*Bl; *Re-*(*1-f*)*Cl; f*Rp + R-Cp	R0-f*Bl + (1*-f*)*Br; -Be-f*Cl; f*Rp -Bs-Cp	Supervision	The public
		*R0 + R-C;* *Re;* *Rs*	*R0;* *-Be;* *-Bs*	No supervision	

#### 4.1.2 Model formulation

The different strategies’ expected earnings and average earnings of enterprise, the government, and the public can be calculated using the payoff matrix of the three-subject game in Table 2; then, we can construct the replicator dynamic equation 1 of each subject.

Let the expected earnings of “green technology innovation,” “traditional technology,” and average expected earnings for enterprises are, respectively, *C1*, *C2*, and *Cx;* they are calculated as follows:

$$\begin{array}{l} C_{1}=y*z*\left(R_{0}+R+C_{b}-C+B_{r}\right)+y*\left(1-z\right)*\\ \left(R_{0}+R+C_{b}-C\right)+\left(1-y\right)*z*\left(R_{0}+R-C+f*B_{r}-\left(1-f\right)*B_{l}\right)\\ +\left(1-y\right)*\left(1-z\right)*\left(R_{0}+R-C\right) \end{array}$$

$$\begin{array}{l} C_{2}=y*z*\left(R_{0}-R_{b}-B_{l}-C_{e}\right)+y*\left(1-z\right)*\left(R_{0}-R_{b}\right)+\left(1-y\right)*\\ z*\left(R_{0}-f*B_{l}+\left(1-f\right)*B_{r}\right)+\left(1-y\right)*\left(1-z\right)*\left(R_{0}\right) \end{array}$$

$$C_{x}=x*C_{1}+\left(1-x\right)*C_{2}$$

The replicator dynamic equation of enterprise strategy can be obtained as follows:

(1) $$F\left(x\right)=\frac{d\left(x\right)}{d\left(t\right)}=x*\left(C_{1}-C_{x}\right)$$

$$=x*\left(1-x\right)*\left[\begin{array}{l} y*\left(R_{b}+C_{b}\right)+z*\left(\begin{array}{l} f*B_{l}-\left(1-f\right)*\\ B_{l}+f*B_{r}-\left(1-f\right)*B_{r} \end{array}\right)\\ +y**\left(\begin{array}{l} B_{r}+B_{l}+\left(1-f\right)*B_{r}-f*\\ B_{r}+\left(1-f\right)*B_{l}-f*B_{l}+C_{e} \end{array}\right)+R-C \end{array}\right]$$

Similarly, let the expected earnings of “regulation,” “no regulation,” and average expected earnings for the government are, respectively, *G1*, *G2*, and *Gy;* they are calculated as follows:

$$\begin{array}{l} G_{1}=x*z*\left(C_{r}+R_{e}-C_{b}-C_{s}\right)+x*\left(1-z\right)*\left(R_{e}-C_{b}\right)\\ +\left(1-x\right)*z*\left(R_{b}-C_{s}-B_{e}\right)+\left(1-x\right)*\left(1-z\right)*\left(R_{b}-B_{e}\right) \end{array}$$

$$\begin{array}{l} G_{2}=x*z*\left(R_{e}-\left(1-f\right)*C_{l}\right)+x*\left(1-z\right)*R_{e}+\left(1-x\right)\\ {}*z*\left(-B_{e}-f*C_{l}\right)+\left(1-x\right)*\left(1-z\right)*\left(-B_{e}\right) \end{array}$$

$$G_{y}=y*G_{1}+\left(1-y\right)*G_{2}$$

The replicator dynamic equation of government strategy can be obtained as follows:

(2) $$F\left(y\right)=\frac{d\left(y\right)}{d\left(t\right)}=y*\left(G_{1}-G_{y}\right)$$

$$=y*\left(1-y\right)*\left[\begin{array}{l} x*\left(-C_{b}-R_{b}\right)+z*\left(\;C_{l}*f-C_{s}\right)\\ +x*z*\left(C_{r}+\left(1-f\right)*C_{l}-f*C_{l}\right)+R_{b} \end{array}\;\right]$$

Similarly, let *P1*, *P2*, and *Pz* represent, respectively, the expected earnings of “supervision,” “no supervision,” and average expected earnings for the public; they are calculated as follows:

$$\begin{array}{l} P_{1}=x*y*\left(R_{s}+C_{s}+R_{p}-C_{p}\right)+x*\left(1-y\right)\\ {}*\left(R_{s}+f*R_{p}-C_{p}\right)+\left(1-x\right)*y*\left(C_{s}+R_{p}-B_{s}-C_{p}+C_{e}\right)\\ +\left(1-x\right)*\left(1-y\right)*\left(f*R_{p}-B_{s}-C_{p}\right) \end{array}$$

$$\begin{array}{l} P_{2}=x*y*R_{s}+x*\left(1-y\right)*R_{s}+\left(1-x\right)*y*\left(-B_{s}\right)+\left(1-x\right)\\ \;*\left(1-y\right)*\left(-B_{s}\right) \end{array}$$

$$P_{z}=z*P_{1}+\left(1-z\right)*P_{2}$$

The replicator dynamic equation of public strategy can be obtained as follows:

(3) $$F\left(z\right)=\frac{d\left(z\right)}{d\left(t\right)}=z*\left(P_{1}-P_{z}\right)$$

$$=z*\left(1-z\right)*\left[\begin{array}{l} y*\left(C_{s}+C_{e}+\left(1-f\right)*R_{p}\right)\\ -x*y*C_{e}-C_{p}+R_{p}*f \end{array}\right]$$

### 4.2 Evolutionary game model analysis

#### 4.2.1 Evolutionary stability of relevant subjects

##### 4.2.1.1 Stability of enterprise

By differentiating with respect to *x* of (*x*), we can obtain the following:

$$\begin{array}{l} \frac{dF\left(x\right)}{dx}=\\ \left(1-2\;x\right)*\left[\begin{array}{l} y*\left(R_{b}+C_{b}\right)+z*\left(\begin{array}{l} f*B_{l}-\left(1-f\right)*B_{l}+\\ f*B_{r}-\left(1-f\right)*B_{r} \end{array}\right)\\ +y*z*\left(\begin{array}{l} B_{r}+B_{l}+\left(1-f\right)*B_{r}-f*B_{r}\\ +\left(1-f\right)*B_{l}-f*B_{l}+C_{e} \end{array}\right)+R-C \end{array}\right] \end{array}$$

make $L\left(y,z,f\right)=\left[\begin{array}{l} y*\left(R_{b}+C_{b}\right)+z*\left(\begin{array}{l} f*B_{l}-\left(1-f\right)\\ {}*B_{l}+f*B_{r}-\left(1-f\right)*B_{r} \end{array}\right)\\ +y*z*\left(\begin{array}{l} B_{r}+B_{l}+\left(1-f\right)*B_{r}-f*B_{r}\\ +\left(1-f\right)*B_{l}-f*B_{l}+C_{e} \end{array}\right)+R-C \end{array}\right]$

From the stability of the replicator dynamic equation, it can be seen that *x*, as a stabilization strategy, should satisfy $F\left(x,y,z\right)$ =0, and $\frac{dF\left(x\right)}{dx}<0$ (37).

When $L\left(y,z,f\right)=0$ , $y=y^{*}=\frac{C-R-z*\left(\begin{array}{l} f*B_{l}-\left(1-f\right)\\ {}*B_{l}+f*B_{r}-\left(1-f\right)*B_{r} \end{array}\right)}{R_{b}+C_{b}+z*\left(\begin{array}{l} B_{r}+B_{l}+\left(1-f\right)*B_{r}-f\\ {}*B_{r}+\left(1-f\right)*B_{l}-f*B_{l}+C_{e} \end{array}\right)}\text{,}$ $F\left(x\right)\text{≡}0$ , which means that all points on the X-axis are in a stable state, and the strategy choice of the enterprise does not change with time (38).

When $\begin{array}{l} \;\mathrm{When}\;L\left(y,z,f\right)\neq 0，\\ y\neq \frac{C-R-z*\left(\begin{array}{l} f*B_{l}-\left(1-f\right)\\ {}*B_{l}+f*B_{r}-\left(1-f\right)*B_{r} \end{array}\right)}{R_{b}+C_{b}+z*\left(\begin{array}{l} B_{r}+B_{l}+\left(1-f\right)*B_{r}-f\\ {}*B_{r}+\left(1-f\right)*B_{l}-f*B_{l}+C_{e} \end{array}\right)}\text{,} \end{array}$ at this time the stable state of the enterprise needs to be discussed by case.

Corollary 1: If the probability of the government choosing the “regulation” strategy is low, the probability of the enterprise choosing the “green technology innovation” option is steady at 0. On the contrary, if the probability of the government selecting the “regulation” strategy is high, the probability of the enterprise choosing the “green technology innovation” option remains steady at 1.

Proof: When $\frac{\partial L\left(y,z,f\right)}{\partial y}=0,f=1+\frac{R_{b}+C_{b}}{2*z*\left(B_{l}+B_{r}\right)}>1\text{;}$ when $f<1+\frac{R_{b}+C_{b}}{2*z*\left(B_{l}+B_{r}\right)},\frac{\partial L\left(y,z,f\right)}{\partial y}$ >0. $L\left(y,z,f\right)$ is an increasing function. Thus, when $0<y<\frac{C-R-z*\left(\begin{array}{l} f*B_{l}-\left(1-f\right)\\ {}*B_{l}+f*B_{r}-\left(1-f\right)*B_{r} \end{array}\right)}{R_{b}+C_{b}+z*\left(\begin{array}{l} B_{r}+B_{l}+\left(1-f\right)*B_{r}-f\\ {}*B_{r}+\left(1-f\right)*B_{l}-f*B_{l}+C_{e} \end{array}\right)}\text{,}$ $L\left(y,z,f\right)<0$ , $\frac{dF\left(x\right)}{dx}{|}_{x=0}<0$ , $\frac{dF\left(x\right)}{dx}{|}_{x=1}>0$ , *x* = 0 is the evolutionary stable point; when $\frac{C-R-z*\left(\begin{array}{l} f*B_{l}-\left(1-f\right)\\ {}*B_{l}+f*B_{r}-\left(1-f\right)*B_{r} \end{array}\right)}{R_{b}+C_{b}+z*\left(\begin{array}{l} B_{r}+B_{l}+\left(1-f\right)*B_{r}-f\\ {}*B_{r}+\left(1-f\right)*B_{l}-f*B_{l}+C_{e} \end{array}\right)}<y<1\text{,}$ $L\left(y,z,f\right)>0$ , $\frac{dF\left(x\right)}{dx}{|}_{x=0}>0$ , $\frac{dF\left(x\right)}{dx}{|}_{x=1}<0$ , x =1 is the evolutionary stable point.

To sum up, the reaction function of the probability of the enterprise choosing “green technology innovation” strategy *x* is as follows:

$$x=\left\{\begin{array}{l} 0\;\mathrm{if}<y<\frac{C-R-z*\left(\begin{array}{l} f*B_{l}-\left(1-f\right)\\ {}*B_{l}+f*B_{r}-\left(1-f\right)*B_{r} \end{array}\right)}{R_{b}+C_{b}+z*\left(\begin{array}{l} B_{r}+B_{l}+\left(1-f\right)*B_{r}-f\\ {}*B_{r}+\left(1-f\right)*B_{l}-f*B_{l}+C_{e} \end{array}\right)}\\ \left[0,1\right]\;\mathrm{if}\;y<\frac{C-R-z*\left(\begin{array}{l} f*B_{l}-\left(1-f\right)\\ {}*B_{l}+f*B_{r}-\left(1-f\right)*B_{r} \end{array}\right)}{R_{b}+C_{b}+z*\left(\begin{array}{l} B_{r}+B_{l}+\left(1-f\right)*B_{r}-f\\ {}*B_{r}+\left(1-f\right)*B_{l}-f*B_{l}+C_{e} \end{array}\right)}\\ 1\;\mathrm{if}\;\frac{C-R-z*\left(\begin{array}{l} f*B_{l}-\left(1-f\right)\\ {}*B_{l}+f*B_{r}-\left(1-f\right)*B_{r} \end{array}\right)}{R_{b}+C_{b}+z*\left(\begin{array}{l} B_{r}+B_{l}+\left(1-f\right)*B_{r}-f\\ {}*B_{r}+\left(1-f\right)*B_{l}-f*B_{l}+C_{e} \end{array}\right)}<y<\;0 \end{array}\right\}$$

**Figure 2:**
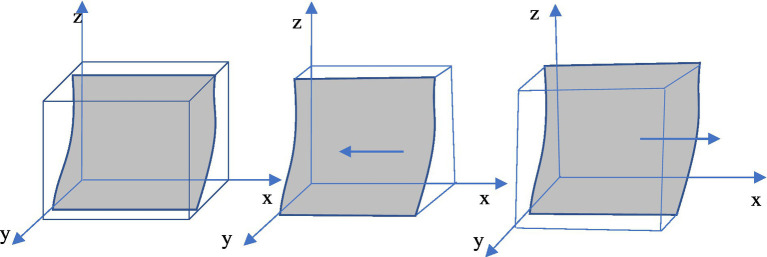
Dynamic evolution trend of enterprise strategy.

At this time, the dynamic evolution trend of the enterprise “green technology innovation” strategy is shown in Figure 2.

Corollary 1 demonstrates that under government regulation, media reports are authentic and reliable. Hence, while the government prefers to regulate, enterprise tends to innovate in green technologies. This is due to the fact that when an enterprise chooses green technology innovation, it can benefit from both government regulation (R&D awards) and media coverage (reputation awards); however, when enterprise opts for traditional technology production, it will face severe penalties from government regulation (fines) and media coverage (reputational damage). On the other hand, if the government chooses not to regulate, enterprise will continue to employ traditional technology with lower production costs.

##### 4.2.1.2 Stability of the government

By differentiating with respect to y of (𝑦), we can obtain the following:

$$\frac{dF\left(y\right)}{\mathrm{dy}}=\left(1-2y\right)*\left[\begin{array}{l} x*\left(-C_{b}-R_{b}\right)+z*\left(\;C_{l}*f-C_{s}\right)+x\\ {}*z*\left(C_{r}+\left(1-f\right)*C_{l}-f*C_{l}\right)+R_{b} \end{array}\right]$$

$$\mathrm{Make}M\left(x,z,f\right)=\left[\begin{array}{l} x*\left(-C_{b}-R_{b}\right)+z*\left(\;C_{l}*f-C_{s}\right)\\ +x*z*\left(C_{r}+\left(1-f\right)*C_{l}-f*C_{l}\right)+R_{b} \end{array}\right]$$

As a stable strategy, *y* should satisfy $F\left(y\right)=0$ , and $\frac{dF\left(y\right)}{\mathrm{dy}}<0$ .

When $M\left(x,z,f\right)=0$ , $x=x^{*}=\frac{-R_{b}-z*\;\left(C_{l}*f-C_{s}\right)}{z*\left(C_{r}+\left(1-f\right)*C_{l}-f*C_{l}\right)-\left(C_{b}+R_{b}\right)}$ , $F\left(y\right)\text{≡}0$ , which means that all points on the Y-axis are in a stable state, and the strategy choice of the government does not change with time.

When $\begin{array}{l} M\left(x,z,f\right)\neq 0，x=x^{*}\\ \neq \frac{-R_{b}-z*\;\left(C_{l}*f-C_{s}\right)}{z*\left(C_{r}+\left(1-f\right)*C_{l}-f*C_{l}\right)-\left(C_{b}+R_{b}\right)} \end{array}$ . At this time, the stable state of the government needs to discuss *f* in different cases.

Corollary 2: Under the case of the probability of media reporting correctly is high. If the probability of the enterprise adopting “green technology innovation” strategy is low, the probability of the government choosing “regulation” is stable at 1. On the contrary, if the probability of the enterprise choosing “green technology innovation” is high, the probability of the government choosing “regulation” is stable at 0.

Proof: When $\frac{\partial M\left(xz,f\right)}{\partial x}$ =0, $f=\frac{1}{2}+\frac{z*C_{r}-\left(C_{b}+R_{b}\right)}{2*z*C_{l}}$ . When $f>\frac{1}{2}+\frac{z*C_{r}-\left(C_{b}+R_{b}\right)}{2*z*C_{l}}$ , $\frac{\partial M\left(x,z,f\right)}{\partial x}<0$ . $M\left(x,z,f\right)$ is a subtraction function. Thus, when $0<x<\frac{-R_{b}-z*\;\left(C_{l}*f-C_{s}\right)}{z*\left(C_{r}+\left(1-f\right)*C_{l}-f*C_{l}\right)-\left(C_{b}+R_{b}\right)}$ , $M\left(x,z,f\right)>0$ , $\frac{dF\left(y\right)}{\mathrm{dy}}{|}_{y=0}>0$ , $\frac{dF\left(y\right)}{\mathrm{dy}}{|}_{y=1}<0$ , *y* = 1 is the evolutionary stable point; when $\frac{-R_{b}-z*\;\left(C_{l}*f-C_{s}\right)}{z*\left(C_{r}+\left(1-f\right)*C_{l}-f*C_{l}\right)-\left(C_{b}+R_{b}\right)}<x<1$ , $M\left(x,z,f\right)<0,\frac{dF\left(y\right)}{\mathrm{dy}}{|}_{y=0}<0$ , $\frac{dF\left(y\right)}{\mathrm{dy}}{|}_{y=1}>0$ , y = 0 is the evolutionary stable point.

To sum up, when $f>\frac{1}{2}+\frac{z*C_{r}-\left(C_{b}+R_{b}\right)}{2*z*C_{l}}$ , the reaction function of the probability of the government choosing “regulation” strategy *y* is as follows:

$$y=\left\{\begin{array}{cc} 1 & \mathrm{if}\;0<x<\frac{-R_{b}-z*\;\left(C_{l}*f-C_{s}\right)}{z*\left(C_{r}+\left(1-f\right)*C_{l}-f*C_{l}\right)-\left(C_{b}+R_{b}\right)}\\ \left[0,1\right]\; & \mathrm{if}\;x=\frac{-R_{b}-z*\;\left(C_{l}*f-C_{s}\right)}{z*\left(C_{r}+\left(1-f\right)*C_{l}-f*C_{l}\right)-\left(C_{b}+R_{b}\right)}\\ 0 & \mathrm{if}\;\frac{-R_{b}-z*\;\left(C_{l}*f-C_{s}\right)}{z*\left(C_{r}+\left(1-f\right)*C_{l}-f*C_{l}\right)-\left(C_{b}+R_{b}\right)}<x<1 \end{array}\right\}$$

**Figure 3:**
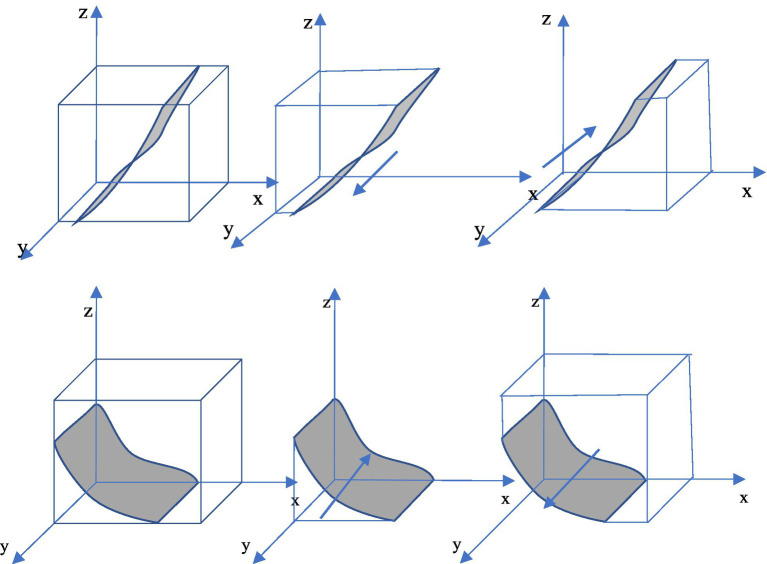
Dynamic evolution trend of government strategy.

At this time, the dynamic evolution trend of government “regulation” strategy is shown in Figure 3.

Corollary 2 shows that under a rational and fair new media environment, media reports emphasize on facts and are more authentic and reliable. At that time, if enterprise gains economic benefits at the expense of the environment and chooses to employ traditional technology, the government would actively regulate to fulfill its responsibilities and avoid reputation damages; however, if enterprise adopts green technology innovation, the government will reduce the intensity of regulation because it can obtain good environmental benefits without doing anything.

Corollary 3: Under the case of the probability of media reporting correctly is low. If the probability of the enterprise adopting “green technology innovation” strategy is low, the probability of the government choosing “regulation” strategy is stable at 0. On the contrary, if the probability of enterprise green technology innovation is high, the probability of government regulation is stable at 1.

Proof: When $f<\frac{1}{2}+\frac{z*C_{r}-\left(C_{b}+R_{b}\right)}{2*z*C_{l}}$ , $\frac{\partial M\left(x,z,f\right)}{\partial x}>0，M\left(x,z,f\right)$ is an increasing function. Thus, when $0<x<\frac{-R_{b}-z*\;\left(C_{l}*f-C_{s}\right)}{z*\left(C_{r}+\left(1-f\right)*C_{l}-f*C_{l}\right)-\left(C_{b}+R_{b}\right)}$ , $M\left(x,z,f\right)<0,\frac{dF\left(y\right)}{\mathrm{dy}}{|}_{y=0}<0$ , $\frac{dF\left(y\right)}{\mathrm{dy}}{|}_{y=1}>0$ , *y* = 0 is the evolutionary stable point; when $\frac{-R_{b}-z*\;\left(C_{l}*f-C_{s}\right)}{z*\left(C_{r}+\left(1-f\right)*C_{l}-f*C_{l}\right)-\left(C_{b}+R_{b}\right)}<x<1$ , $M\left(x,z,f\right)>0,\frac{dF\left(y\right)}{\mathrm{dy}}{|}_{y=0}>0$ , $\frac{dF\left(y\right)}{\mathrm{dy}}{|}_{y=1}<0$ , *y* = 1 is the evolutionary stable point.

To sum up, when $f<\frac{1}{2}+\frac{z*C_{r}-\left(C_{b}+R_{b}\right)}{2*z*C_{l}}$ , the reaction function of the probability of the government choosing “regulation” strategy *y* is as follows:

$$y=\left\{\begin{array}{cc} 0 & \mathrm{if}\;0<x<\frac{-R_{b}-z*\;\left(C_{l}*f-C_{s}\right)}{z*\left(C_{r}+\left(1-f\right)*C_{l}-f*C_{l}\right)-\left(C_{b}+R_{b}\right)}\\ \left[0,1\right]\; & \mathrm{if}\;x=\frac{-R_{b}-z*\;\left(C_{l}*f-C_{s}\right)}{z*\left(C_{r}+\left(1-f\right)*C_{l}-f*C_{l}\right)-\left(C_{b}+R_{b}\right)}\\ 1 & \mathrm{if}\;\frac{-R_{b}-z*\;\left(C_{l}*f-C_{s}\right)}{z*\left(C_{r}+\left(1-f\right)*C_{l}-f*C_{l}\right)-\left(C_{b}+R_{b}\right)}<x<1 \end{array}\right\}$$

At this time, the dynamic evolution trend of government “regulation” strategy is shown in Figure 3.

Corollary 3 shows that the government’s behavior in a media environment with distorted reports is completely opposite to that in a fair media environment. When an enterprise carries out green technology innovation, the government tends to choose regulation to avoid serious reputation loss. This situation is extremely unfavorable to social and economic development, which may cause the mutual trust between enterprise and the government to be lost and even cause unnecessary social unrest. Therefore, it is extremely necessary to create a rational and fair media environment.

##### 4.2.1.3 Stability of the public

By differentiating with respect to *z* of (*z*), we can obtain the following:

$$\frac{dF\left(z\right)}{dz}=\left(1-2z\right)*\left[\begin{array}{l} y*\left(C_{s}+C_{e}+\left(1-f\right)*R_{p}\right)\\ -x*y*C_{e}-C_{p}+R_{p}*f \end{array}\right]$$

$$\mathrm{Make}\frac{dF\left(z\right)}{dz}=\left(1-2z\right)*\left[\begin{array}{l} y*\left(C_{s}+C_{e}+\left(1-f\right)*R_{p}\right)\\ -x*y*C_{e}-C_{p}+R_{p}*f \end{array}\right]$$

As a stable strategy, *z* should satisfy $F\left(z\right)=0$ , and $\frac{dF\left(z\right)}{dz}<0.$

When $N\left(x,y,f\right)=0$ , $x=x^{**}=\frac{y*\left(C_{s}+C_{e}+\left(1-f\right)*R_{p}\right)-C_{p}+R_{p}*f}{y*C_{e}}$ , $F\left(z\right)\text{≡}0$ , which means that all points on the Z-axis are in a stable state, and the strategy choice of the public does not change with time.

When $\begin{array}{l} \;N\left(x,y,f\right)\neq 0，x=x^{**}\\ \neq \frac{y*\left(C_{s}+C_{e}+\left(1-f\right)*R_{p}\right)-C_{p}+R_{p}*f}{y*C_{e}}\text{,} \end{array}$ at this time, the stable state of the public needs to discuss *f* in different cases. By x>0 , it can be obtained $f>\frac{C_{p}-y*\left(C_{s}+C_{e}+R_{p}\right)}{\left(1-y\right)*R_{p}}$ 。

Corollary 4: Under the case of the probability of media reporting correctly is high. If the probability of the enterprise adopting “green technology innovation” strategy is low, the probability of the public choosing “supervision” strategy is stable at 1. On the contrary, if the probability of enterprise green technology innovation is high, then the probability of public supervision is stable at 0.

Proof: When $f>\frac{C_{p}-y*\left(C_{s}+C_{e}+R_{p}\right)}{\left(1-y\right)*R_{p}}$ , $\frac{\partial N\left(x,y,f\right)}{\partial x}<0$ , $N\left(x,y,f\right)$ is an increasing function. Then, the proof process is based on Corollary 2, which is omitted here, that is, when $f>\frac{C_{p}-y*\left(C_{s}+C_{e}+R_{p}\right)}{\left(1-y\right)*R_{p}}\text{,}$ the reaction function of the probability of the public choosing “supervision” strategy *z* is as follows:

$$z=\left\{\begin{array}{cc} 1 & \mathrm{if}\;0<x<\frac{y*\left(C_{s}+C_{e}+\left(1-f\right)*R_{p}\right)-C_{p}+R_{p}*f}{y*C_{e}}\\ \left[0,1\right]\; & \;ifx=\frac{y*\left(C_{s}+C_{e}+\left(1-f\right)*R_{p}\right)-C_{p}+R_{p}*f}{y*C_{e}}\\ 0 & \mathrm{if}\;\frac{y*\left(C_{s}+C_{e}+\left(1-f\right)*R_{p}\right)-C_{p}+R_{p}*f}{y*C_{e}}<x<1 \end{array}\right\}$$

**Figure 4:**
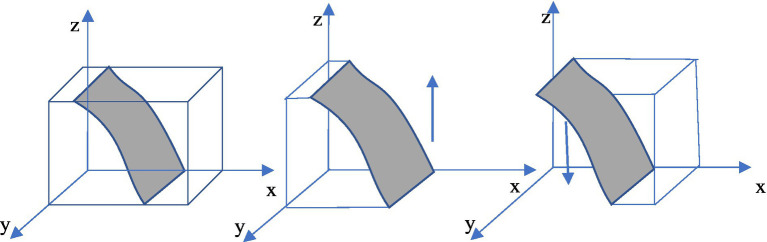
Dynamic evolution trend of public strategy.

At this time, the dynamic evolution trend of public supervision is shown in Figure 4.

Corollary 4 shows that under a rational and fair new media environment, media reports are more authentic and reliable. Hence, while enterprise chooses “green technology innovation” strategy, the public can obtain high environmental benefits even if it does nothing. In this case, the public tends to choose not to supervise. On the contrary, when an enterprise chooses “traditional technology” strategy, the public will suffer serious environmental losses and choose to supervise.

From the above analysis, it can be found that the behavioral strategy of enterprise, the government, or the public is not directly made by an independent subject but depends on the strategic choices of the other two subjects. Meanwhile, the accuracy of media reports plays an important role in this process. This also reflects the necessity of studying the cooperation among enterprise, the government, and the public in the new media environment for the formation of green technology innovation.

#### 4.2.2 Evolutionary stability of green technology innovation system

In combination with Equations 1–3, the replicator dynamic equation of green technology innovation system under a new media environment can be determined as shown in Equation 4:

$$\begin{array}{l} F\left(x\right)=\frac{d\left(x\right)}{d\left(t\right)}=x*\left(C_{1}-C_{x}\right)=x*\left(1-x\right)*\\ \;\left[\begin{array}{l} y*\left(R_{b}+C_{b}\right)+z*\left(\begin{array}{l} f*B_{l}-\left(1-f\right)\\ {}*B_{l}+f*B_{r}-\left(1-f\right)*B_{r} \end{array}\right)\\ +y*z*\left(\begin{array}{l} B_{r}+B_{l}+\left(1-f\right)*B_{r}-f\\ {}*B_{r}+\left(1-f\right)*B_{l}-f*B_{l}+C_{e} \end{array}\right)+R-C \end{array}\right] \end{array}$$

(4) $$\begin{array}{l} F\left(y\right)=\frac{d\left(y\right)}{d\left(t\right)}=y*\left(G_{1}-G_{y}\right)=y*\left(1-y\right)\\ \;*\left[\begin{array}{l} x*\left(-C_{b}-R_{b}\right)+z*\left(\;C_{l}*f-C_{s}\right)+x\\ {}*z*\left(C_{r}+\left(1-f\right)*C_{l}-f*C_{l}\right)+R_{b} \end{array}\;\right] \end{array}$$

$$\begin{array}{l} \;F\left(z\right)=\frac{d\left(z\right)}{d\left(t\right)}=z*\left(P_{1}-P_{z}\right)=z*\left(1-z\right)*\\ \;\left[y*\left(C_{s}+C_{e}+\left(1-f\right)*R_{p}\right)-x*y*C_{e}-C_{p}+R_{p}*f\right] \end{array}$$

The key to evolutionary equilibrium analysis is to analyze the evolutionary equilibrium strategy of the system ② (39). Make *F(x)* = 0, *F(y)* = 0, and *F(z)* = 0, eight pure strategy equilibrium points of the system can be obtained (40): *E_1_(0,0,0)*, *E_2_(1,0,0)*, *E_3_(0,1,0)*, *E_4_(0,0,1)*, *E_5_(1,1,0)*, *E_6_(1,0,1)*, *E_7_(0,1,1)*, *E_8_(1,1,1)*.

According to Friedman et al. (41), the stability of the strategy equilibrium point can be obtained by analyzing the characteristics of its Jacobian matrix. Therefore, the partial derivatives of *F(x)*, *F(y),* and *F(z)* with respect to *x*, *y,* and *z* could be obtained as follows:

$$J=\left[\begin{array}{ccc} \frac{\partial F\left(x\right)}{\partial x} & \frac{\partial F\left(x\right)}{\partial y} & \frac{\partial F\left(x\right)}{\partial z}\\ \frac{\partial F\left(y\right)}{\partial x} & \frac{\partial F\left(y\right)}{\partial y} & \frac{\partial F\left(y\right)}{\partial z}\\ \frac{\partial F\left(z\right)}{\partial x} & \frac{\partial F\left(z\right)}{\partial y} & \frac{\partial F\left(z\right)}{\partial z} \end{array}\right]=\left[\begin{array}{ccc} a_{11} & a_{12} & a_{13}\\ a_{21} & a_{22} & a_{23}\\ a_{31} & a_{32} & a_{33} \end{array}\right]$$

$$a_{11}=\left(1-2\;x\right)*\left[\begin{array}{l} y*\left(R_{b}+C_{b}\right)+z*\left(\begin{array}{l} f*B_{l}-\left(1-f\right)\\ {}*B_{l}+f*B_{r}-\left(1-f\right)*B_{r} \end{array}\right)\\ +y*z*\left(\begin{array}{l} B_{r}+B_{l}+\left(1-f\right)*B_{r}-f\\ {}*B_{r}+\left(1-f\right)*B_{l}-f*B_{l} \end{array}\right)+R-C \end{array}\right]$$

$$\begin{array}{l} a_{12}=x*\left(1-x\right)*[R_{b}+C_{b}+z*\\ \left(B_{r}+B_{l}+\left(1-f\right)*B_{r}-f*B_{r}+\left(1-f\right)*B_{l}-f*B_{l}\;\right] \end{array}$$

$$a_{13}=x*\left(1-x\right)*\left[\begin{array}{l} f*B_{l}-\left(1-f\right)*B_{l}+f*B_{r}-\left(1-f\right)\\ {}*B_{r}+y*\left(\begin{array}{l} B_{r}+B_{l}+\left(1-f\right)*B_{r}-f\\ {}*B_{r}+\left(1-f\right)*B_{l}-f*B_{l} \end{array}\right) \end{array}\;\right]$$

$$a_{21}=y*\left(1-y\right)*\left[\left(-C_{b}-R_{b}\right)+z*\left(C_{r}+\left(1-f\right)*C_{l}-f*C_{l}\right)\;\right]$$

$$a_{22}=\left(1-2y\right)*\left[\begin{array}{l} x*\left(-C_{b}-R_{b}\right)+z*\left(\;C_{l}*f-C_{s}\right)\\ +x*z*\left(C_{r}+\left(1-f\right)*C_{l}-f*C_{l}\right)+R_{b} \end{array}\;\right]$$

$$a_{23}=y*\left(1-y\right)*\;\left[\left(\;C_{l}*f-C_{s}\right)+x*\left(C_{r}+\left(1-f\right)*C_{l}-f*C_{l}\right)\right]$$

$$a_{31}=z*\left(1-z\right)*\left[-y*C_{e}\right]$$

$$a_{32}=z*\left(1-z\right)*\left[\left(C_{s}+C_{e}+\left(1-f\right)*R_{p}\right)-x*C_{e}\right]$$

$$a_{33}=\left(1-2z\right)*\left[\begin{array}{l} y*\left(C_{s}+C_{e}+\left(1-f\right)*R_{p}\right)\\ -x*y*C_{e}-C_{p}+R_{p}*f \end{array}\right]$$

According to *Lyapunov’s first rule* (42), if the pure strategy equilibrium point in the three-subject game of enterprise, the government, and the public is ESS, the characteristic roots of its corresponding Jacobian matrix should be less than 0. The stability analysis of the eight pure strategy equilibrium points is shown in Table 3.

**Table 3:** Stability analysis of the pure strategy equilibrium points.

Equilibrium	λ1	λ2	λ3	Stability conditions
*E1*(0,0,0)	*R-C*	*R_b_* (+)	Rp*f-Cp	Instability point
*E2*(1,0,0)	*C-R*	*-Cb*	Rp*f-Cp	C < R; Rp*f < Cp
*E3*(0,1,0)	*Cb-C + R + Rb* (+)	*-R_b_*	*Ce-Cp + Cs + Rp*	Instability point
*E4*(0,0,1)	R-Br-C-B_l_ + 2*Bl*f + 2*Br*f	Rb-Cs + Cl*f	Cp-Rp*f	R-Br-C-Bl + 2*Bl*f + 2*Br*f < 0; Rb-Cs + Cl*f < 0; Cp-Rp*f < 0
*E5*(1,1,0)	*C-Cb-R-Rb*	*Cb* (+)	*Cs-Cp + Rp*	Instability point
*E5(1,1,0)*	B_l_ + B_r_ + C-R-2*B_l_*f-2*B_r_*f	C_l_-C_b_ + C_r_-C_s_-C_l_*f	C_p_-R_p_*f	B_l_ + B_r_ + C-R-2*B_l_*f-2*B_r_*f < 0; C_l_-C_b_ + C_r_-C_s_-C_l_*f < 0; C_p_-R_p_*f < 0
*E7*(0,1,1)	*Bl + Br-C + Cb + Ce + R + Rb*(+)	Cs-Rb-Cl*f	*Cp-Ce-Cs-Rp*	Instability point
*E_8_(1,1,1)*	*C-B_r_-B_l_-C_b_-C_e_-R-R_b_*	C_b_-C_l_-C_r_ + C_s_ + C_l_*f	*C_p_-C_s_-R_p_*	*C-B_r_-B_l_-C_b_-C_e_-R-R_b_ < 0;* C_b_-C_l_-C_r_ + C_s_ + C_l_*f < 0; *C_p_-C_s_-R_p_ < 0*

Obviously, since the characteristic root *λ2* of the pure policy equilibrium points E_1_(0,0,0) and E_5_(1,1,0) is bigger than 0, these two equilibrium points are unstable points. According to *R* > *C*, the characteristic *λ1* of *E_3_(0,1,0)* and *E_7_(0,1,1)* is positive, so they are not evolutionary equilibrium strategies either. Therefore, we only need to consider the remaining four scenarios. In other words, if the equilibrium points E_2_(1,0,0), E_4_(0,0,1), E_6_(1,0,1), or E_8_(1,1,1) are evolutionary stable strategy, it should meet the following requirements:

(1) when *C* < *R*, -C_b_ < 0 and R_p_*f < C_p_, (1,0,0) is ESS.

(2) when R-B_r_-C-B_l_ + 2*B_l_*f + 2*B_r_*f < 0, R_b_ -C_s_ + C_l_*f < 0 and C_p_-R_p_*f < 0, (0,0,1) is ESS.

(3) when B_l_ + B_r_ + C-R-2*B_l_*f-2*B_r_*f < 0, C_l_ -C_b_ + C_r_ -C_s_ -C_l_*f < 0 and C_p_-R_p_*f < 0, (1,0,1) is ESS.

(4) when *C-B_r_-B_l_-C_b_ -C_e_-R-R_b_ < 0*, C_b_ -C_l_ -C_r_ + C_s_ + C_l_*f < 0 and *C_p_-C_s_-R_p_ < 0,* (1,1,1) is ESS.

## 5 Numerical simulation

The above evolutionary equilibrium analysis shows that the behavioral strategies of enterprise, the government, and the public are not only influenced by their own internal benefits and costs but also influenced by the external new media environment. To further visually demonstrate the strategic choices of enterprises, governments, and the public under the new media environment, this study finally selects heavily polluting enterprises as simulation objects by summarizing a large number of literature, conducting field interviews with enterprises, and listening to expert suggestions. In addition, MATLAB software was used to simulate four possible evolutionary equilibrium strategies of the green technology innovation system in the new media environment to further explore the influence of changes in the values of internal and external parameters on the system evolution trajectory. It should be noted that the initial value of each parameter only reflects the sensitivity relationship between the strategy of the game player and related factors, which is a reflection of the relationship, not the real data in reality.

### 5.1 Numerical simulation of (1,0,0) scenario

When *C* < *R*, -C_b_ < 0 and R_p_*f < C_p_, (1,0,0) is ESS. That is to say, when the incremental benefits of green technology innovation are greater than the innovation costs, enterprise will choose green technology innovation according to the principle of profit maximization. When government regulation needs to give green R&D awards to enterprise, the government chooses no regulation. When the costs of public supervision are greater than the green utility brought by new media, the public will choose no supervision. Under this scenario, point (1,0,0) is an evolutionarily stable strategy, which means that after repeated games, enterprise tends to green technology innovation, the government tends not to regulate, and the public chooses no supervision.

**Figure 5:**
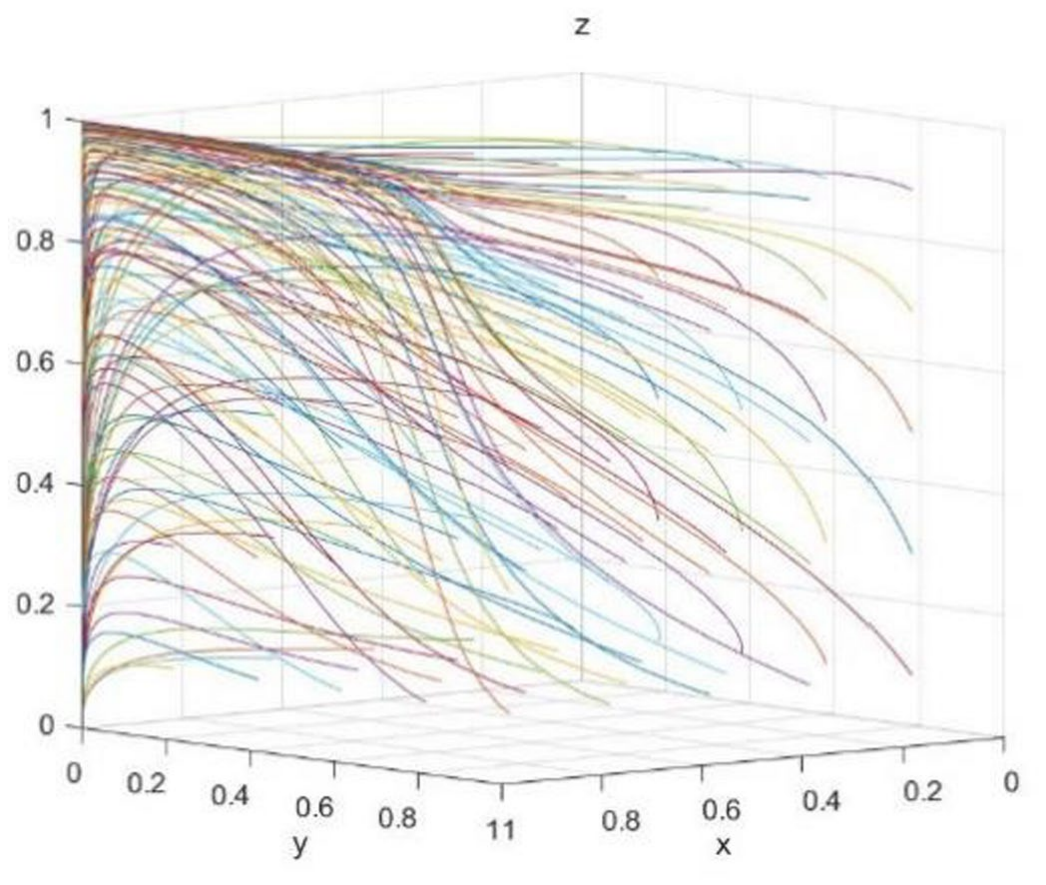
Effect of initial probability on (1,0,0).

**Figure 6:**
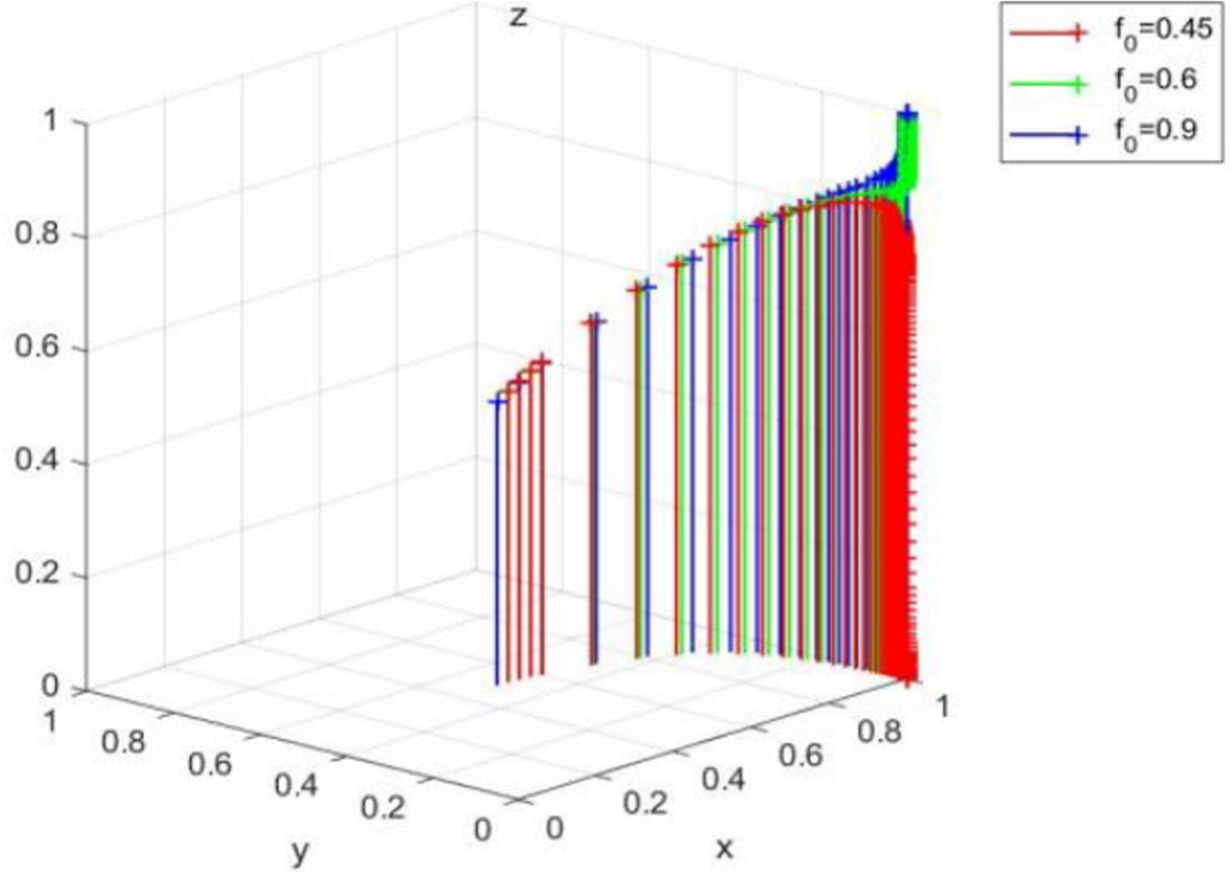
Effect of media report accuracy rate f on (1,0,0).

The evolution path was further clarified by numerical simulation. According to the conditions of (1,0,0) evolution equilibrium, the relevant parameters are set as follows: C*b = 10; Rb = 12; Cs = 20; R0 = 30; R = 11; C = 6; Re = 10; Rs = 12; Be = 12; Bs = 14; Cp = 7; Ce = 8; Cr = 10; Cl = 15; Bl = 18; Br = 14; Rp = 12; f = 0.45.*First, the influence of random initial probability on the evolutionary equilibrium strategy (1,0,0) is verified, which is shown in Figure 5. As shown in the figure, the initial probability values will affect the speed at which enterprise, the government, and the public approach the equilibrium state (1,0,0) but do not affect the result of the system evolution, that is, as long as meet the *C* < *R* and R_p_*f < C_p_ < Cp parameter condition, the three-subject game would evolve to the stable strategy state of (enterprise green technology innovation, the government no regulation, the public does not supervise).

(1,0,0) is an evolutionary equilibrium strategy in a super ideal state that enterprise spontaneously carries out green technology innovation under no government regulation, no public supervision, and no media participation. However, it can be seen from the above theoretical analysis that green technology innovation has double externalities and is difficult to form spontaneously; thus, the equilibrium state is easy to change with the change of external conditions, and it can be inferred from the conditions of equilibrium that the change of the accuracy of new media reports may break this equilibrium. Figure 6 verifies the influence of the change of *f* value (*f* = 0.45, *f* = 0.6, *f* = 0.9) on the evolution equilibrium strategy (1,0,0). It can be seen that, with the increase of *f* until it is beyond the range, enterprise and the government still maintain the original strategy, but the public’s strategy choice changes (Rp*f < Cp is no longer valid), and the equilibrium point of the system will evolve from (1,0,0) to (1,0,1).

It further shows that a fair and just media environment can bring the value guidance of green concept to the public, so as to guide the public to actively participate in the ranks of enterprise behavior supervision.

### 5.2 Numerical simulation of (0,0,1) scenario

When R-B_r_-C-B_l_ + 2*B_l_*f + 2*B_r_*f < 0, R_b_-C_s_ + C_l_*f < 0 and C_p_-R_p_*f < 0, namely $\frac{C_{p}}{R_{p}}<f<\min\left\{\frac{1}{2}+\frac{C-R\;}{2*\left(B_{r}+B_{l}\right)},\;\frac{C_{s}-R_{b}}{C_{l}}\right\}$ , (0,0,1) is ESS, that is, under the media environment, enterprise tends to choose the traditional technology when the benefits of green technology innovation are less than traditional technology; the government is inclined to not regulate when the reputation loss of non-regulation is small, and the green subsidies given to the public are greater than the fines to the traditional technology enterprise of regulation; the public chooses to supervise when the green utilities brought by new media environment are greater than the costs of supervision.

**Figure 7:**
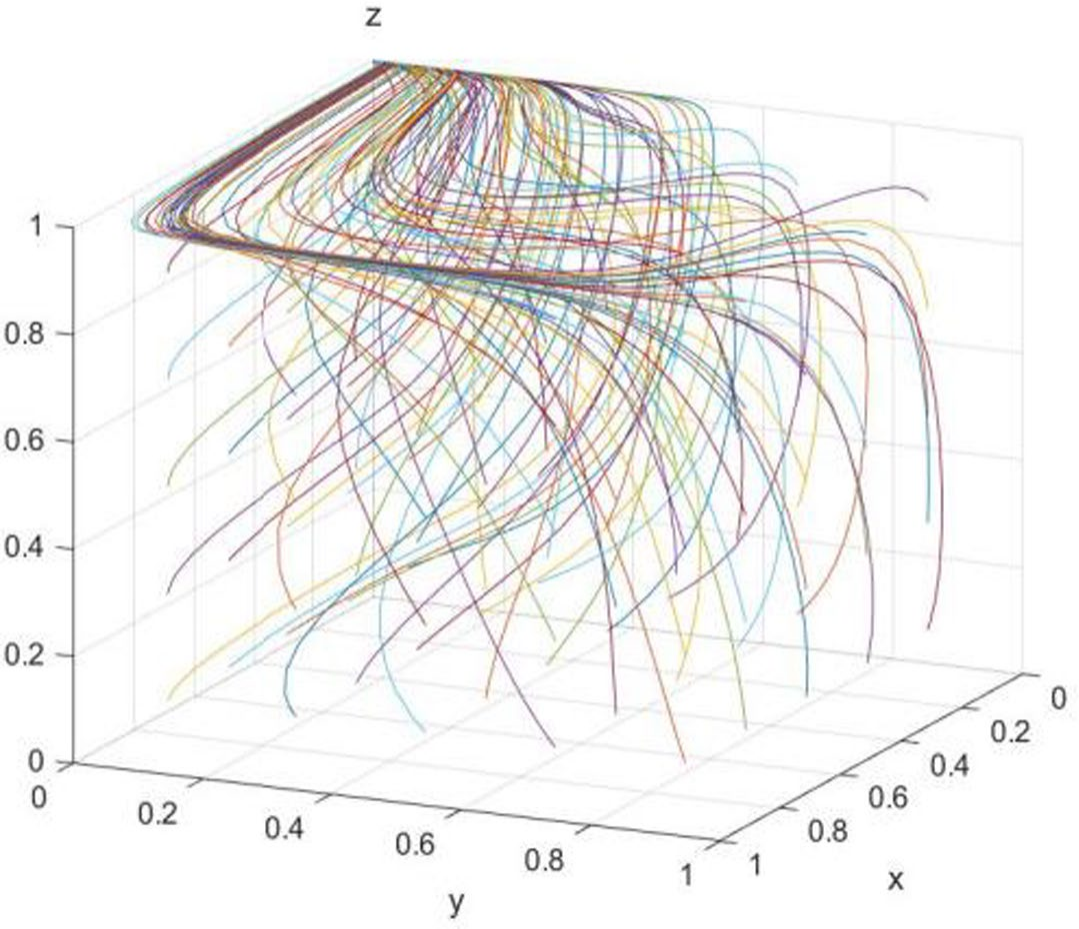
Effect of initial probability on (0,0,1).

The evolution path was further clarified by numerical simulation. According to the conditions of (0,0,1) evolutionarily stable strategy, the relevant parameters are assumed as follows: C*_b_ = 10; R_b_ = 12; C_s_ = 20; R_0_ = 30; R = 7; C = 6; R_e_ = 10; R_s_ = 12; B_e_ = 12; B_s_ = 14; C_p_ = 5; C_e_ = 8; C_r_ = 10; C_l_ = 15; B_l_ = 18; B_r_ = 14; R_p_ = 12; f = 0.45.* First, the influence of random initial probability on the evolutionary equilibrium strategy (0,0,1) is verified, which is shown in Figure 7. It is obvious that the initial probability values will affect the speed of each subject approach to the equilibrium state but do not affect the result of the system evolution, indicating that as long as the parameter conditions of R-B_r_-C-B_l_ + 2*B_l_*f + 2*B_r_*f < 0, R_b_-C_s_ + C_l_*f < 0, and C_p_-R_p_*f < 0 are meet, the three-subject game would evolve to (0,0,1) evolutionary equilibrium strategy.

**Figure 8:**
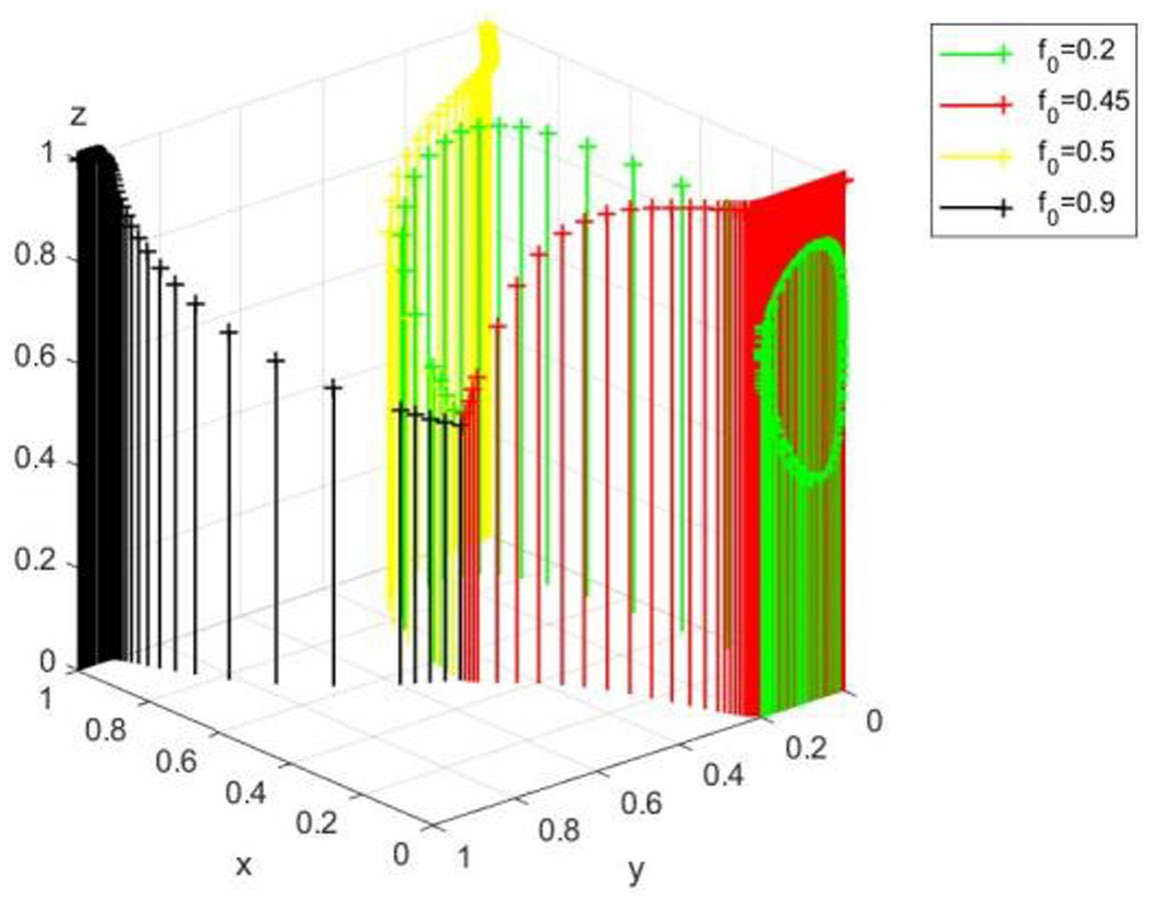
Effect of media report accuracy rate f on (0,0,1).

The ESS of (0,0,1) is ineffective and harmful to the public welfare—the government neglects to regulate and the public supervises actively but cannot reverse enterprise traditional technology behavior. However, as can be seen from the equilibrium satisfying conditions, R-B_r_-C-B_l_ + 2*B_l_*f + 2*B_r_*f < 0, R_b_-C_s_ + C_l_*f < 0 and C_p_-R_p_*f < 0 may no longer hold as the new media environment changes. Figure 8 verifies the influence of the change of *f* value on (0,0,1) when other conditions are unchanged. It can be found that when *f* is too small (*f* = 0.2), C_p_-R_p_*f < 0 is no longer true, and the evolutionary equilibrium strategy of the system evolves from (0,0,1) to (0,0,0). However, as *f* increases (*f* = 0.5, *f* = 0.9), the system may evolve to a better equilibrium state of (1,0,1) or (1,1,1).

It shows that the authenticity of media report is very important. Whether the public chooses to supervise or not depends on the value-leading role of media opinion; media reports can influence corporate profits through reputation mechanism, thus forcing enterprises to change from traditional technology to green technology innovation. A relatively fair new media environment will also increase the reputation losses caused by government’s non-regulation, so that the government will choose to regulation to maintain a good social image.

Further shows that the opinion of new media environment has the important influence to enterprise, government, and the public. A fair media environment can restrict the traditional technology of enterprise and the unregulated behavior of the government. At the same time, the improvement of the correct rate of media reports will promote the public to supervise.

### 5.3 Numerical simulation of (1,0,1) scenario

When B_l_ + B_r_ + C-R-2*B_l_*f-2*B_r_*f < 0, C_l_-C_b_ + C_r_-C_s_-C_l_*f < 0 and C_p_-R_p_*f < 0, namely $\max\left\{\frac{C_{p}}{R_{p}}，\frac{1}{2}+\frac{C-R\;}{2*\left(B_{r}+B_{l}\right)},1-\;\frac{C_{s}+C_{b}-C_{r}}{C_{l}}\right\}<f<1$ , (1,0,1) is ESS, that is to say, under the new media environment, when an enterprise choosing green technology innovation could obtain more benefits than traditional technology, enterprise tends to green technology innovation; when the net benefits of government regulation are negative and the loss values are higher than the reputation losses of no regulation, the government is inclined to not regulate; when the green utilities are greater than supervision costs, the public chooses to supervise. (1,0,1) is a good evolutionary stable strategy that new media reports and public supervision promote enterprise green technology innovation in the absence of government regulation.

**Figure 9:**
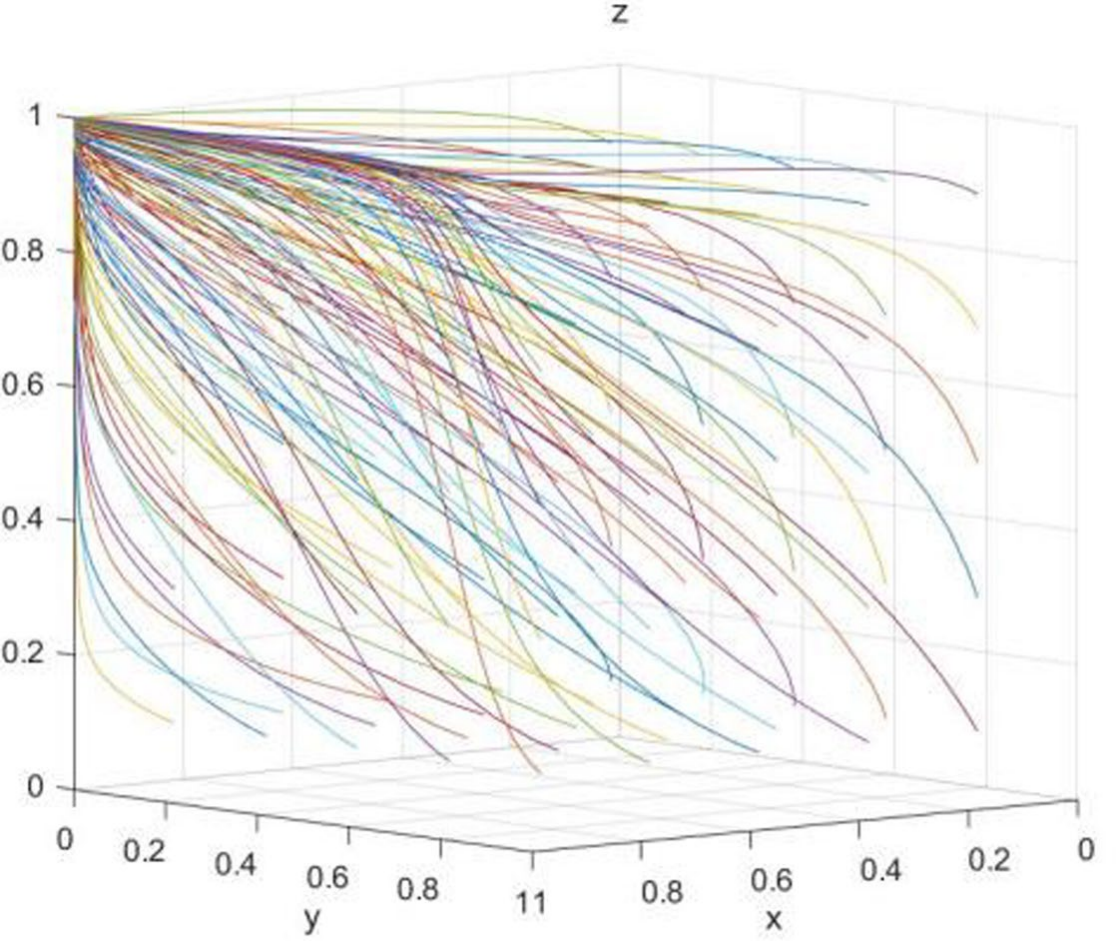
Effect of initial probability on (1,0,1).

**Figure 10:**
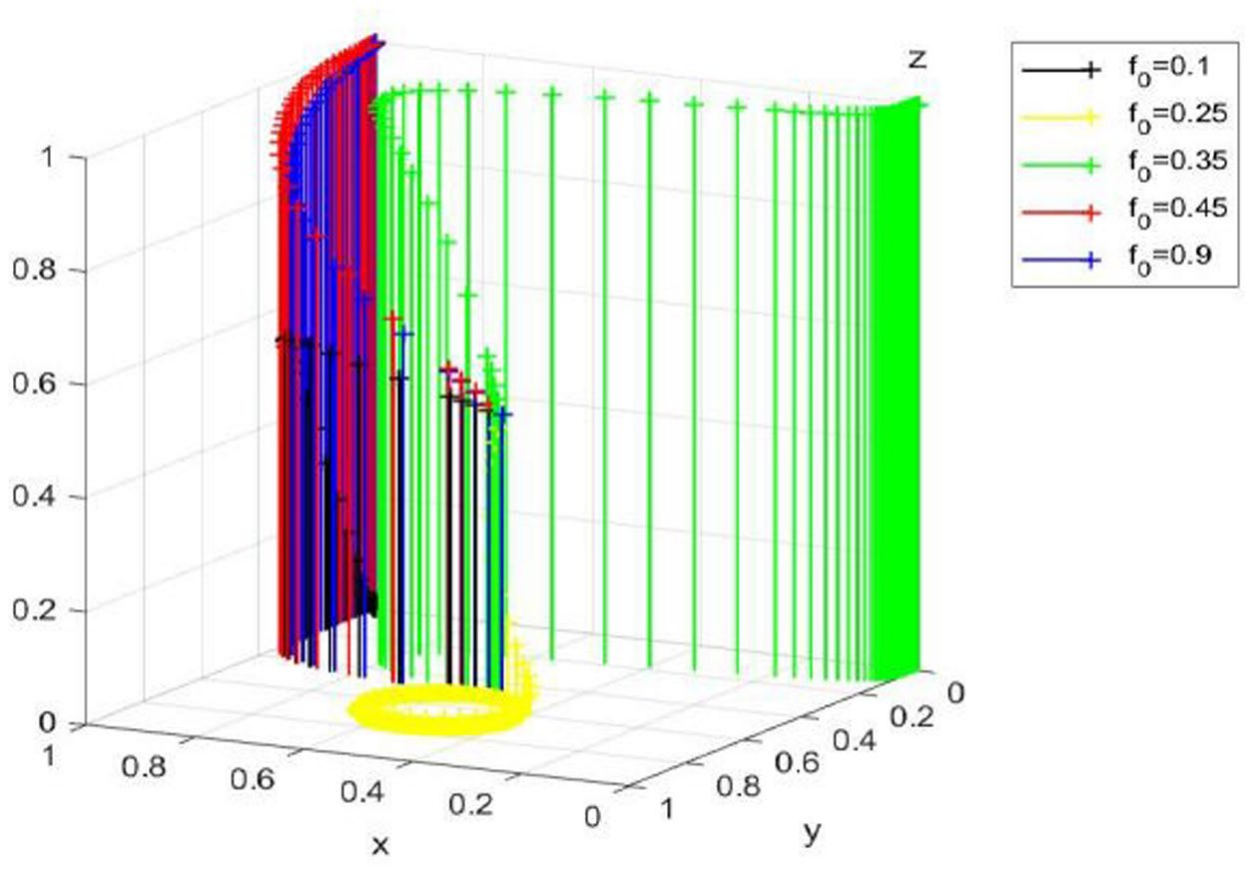
Effect of media report accuracy rate f on (1,0,1).

The evolution path was further clarified by numerical simulation. According to the conditions of (1,0,1) evolutionarily stable strategy, the relevant parameters are set as follows: C*b = 10; Rb = 12; Cs = 20; R0 = 30; R = 11; C = 6; Re = 10; Rs = 12; Be = 12; Bs = 14; Cp = 5; Ce = 8; Cr = 10; Cl = 15; Bl = 18; Br = 14; Rp = 15; f = 0.45*. First, by verifying the influence of random initial probability (Figure 9), it is found that the initial probability only affects the speed of system evolution but not the evolution result. Then, the influence of the accuracy rate *f* of new media reports (Figure 10) is verified. It can be seen that, as *f* gradually increases within the value range (*f* = 0.45, *f* = 0.9), the green technology innovation system evolves to the stable state (1,0,1) at a faster speed. However, as *f* becomes smaller and smaller (*f* = 0.35, *f* = 0.25, *f* = 0.1), Bl + Br + C-R-2*Bl*f-2*Br* f < 0, Cl-Cb + Cr-Cs-Cl* f < 0,and Cp-Rp*f < 0 no longer hold anymore, and the system may evolve to (0,0,1), (0,0,0), or (0,1,0) bad equilibrium strategies.

This indicates that in the process of green technology innovation, the authenticity of new media reports is an important influencing factor. The more trueful the media report is, the easier the evolution stable strategy (enterprise green technology innovation, government non-regulation, public supervision) can be facilitated. However, if the authenticity of media reports declines, the opinion environment that is difficult to distinguish between truths and falsehoods not only confuses the public and disturbs the judgment of the government but also enhances the motivation of enterprise to “pollute first and treat later,” which will have a negative impact on the green technology innovation system.

### 5.4 Numerical simulation of (1,1,1) scenario

When *C-B_r_-B_l_-C_b_-C_e_-R-R_b_ < 0*, C_b_-C_l_-C_r_ + C_s_ + C_l_*f < 0(*0 < f < (-C_b_ + C_l_ + C_r_ + C_s_)/C_l_*), and *C_p_-C_s_-R_p_ < 0,* (1,1,1) is ESS, that is to say, when the profits of enterprise green technology innovation are greater than traditional technology production under the condition of government regulation and public supervision, the difference between government regulation costs and the reputation rewards is less than the reputation losses of no regulation; when the sum of green utilities and government subsidies of public supervision is greater than the supervision costs, the stable strategy of enterprise, the government, and the public is *x* = 1, *y* = 1, and *z* = 1.

**Figure 11:**
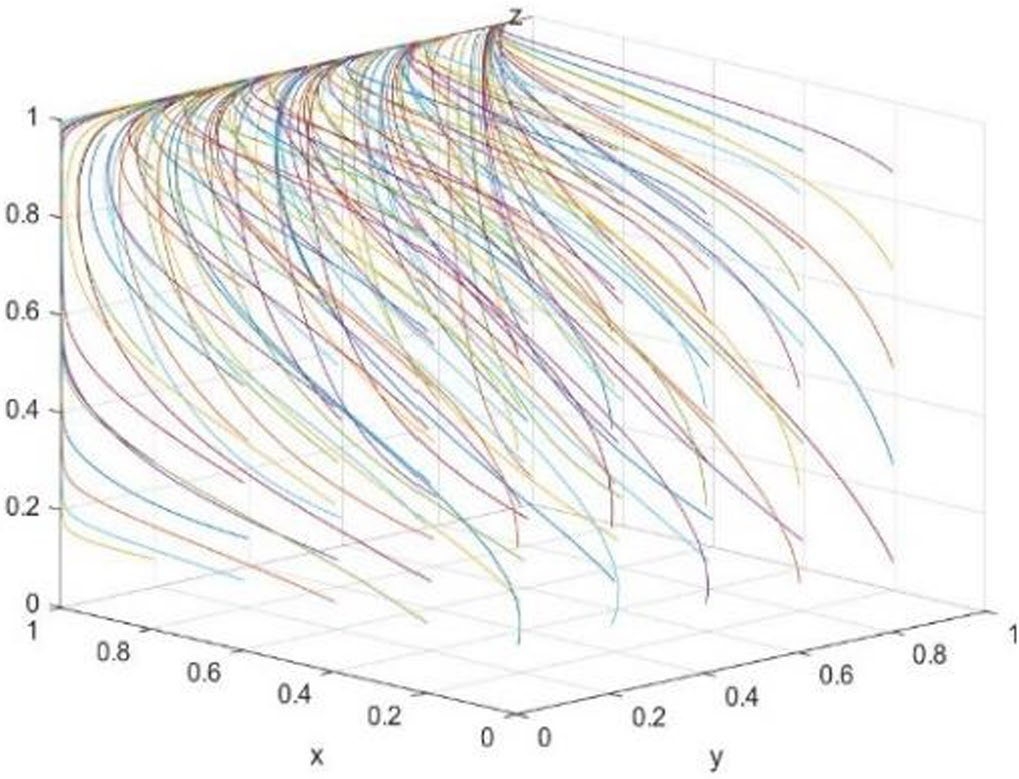
Effect of initial probability on (1,1,1).

**Figure 12:**
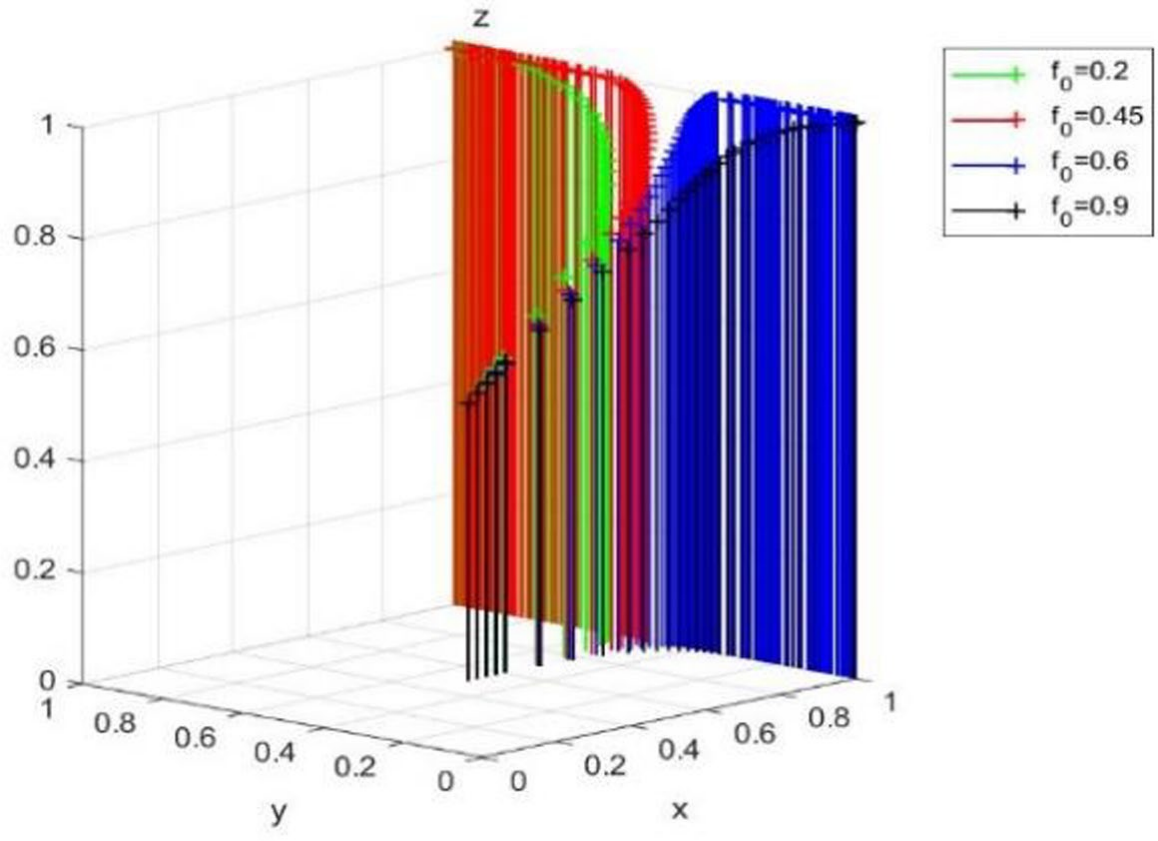
Effect of media report accuracy rate f on (1,1,1).

The evolution path was further clarified by numerical simulation. According to the conditions of (1,1,1) evolutionarily stable strategy, the relevant parameters are assumed as follows: C*b = 10; Rb = 12; Cs = 20; R0 = 30; R = 11; C = 6; Re = 10; Rs = 12; Be = 12; Bs = 14; Cp = 5; Ce = 8; Cr = 20; Cl = 22; Bl = 18; Br = 14; Rp = 12; f = 0.45.* First, by verifying the influence of random initial probability (Figure 11), it is found that the initial probability only affects the speed of system evolution but not the evolution result. Then, the influence of the accuracy rate *f* of new media reports (Figure 12) is explored. Under this condition, the strategy evolution of enterprise or the public is not affected by the correct rate of media reports. Whether enterprise chooses green technology innovation or not depends on the incremental benefits and costs of green technology innovation as well as the influence of media opinions, and the public makes a strategic choice by weighing whether the green utilities could make up for the supervision costs, while the government’s strategy choice will be affected by *f*. When *f* is small (*f* = 0.2, *f* = 0.45), Cb-Cl-Cr + Cs + Cl*f < 0 is still true, indicating that media misreporting increased the reputation losses of the government. When f increases until it goes beyond the range(*f* = 0.6, *f* = 0.9), the reputation losses caused by media misreporting of government non-regulation gradually decrease, while the government regulation costs remain unchanged, the government will change from regulation to non-regulation, and the evolutionary stable strategy of (1,1,1) may eventually evolve to (1,0,1).

Further shows that false media information leads to the government regulation low efficiency or even ineffectiveness and waste regulatory resources. The fair and just new media environment has a certain replacement effect on government regulation, that is, when the public actively participates in supervision and the media reports have a high accuracy rate, even if the government chooses not to regulate, enterprise will still choose to green technology innovation.

## 6 Discussion and conclusion

### 6.1 Discussion

Green technology innovation has recently become a popular research topic. Wang et al. (43) explored the role of environmental regulation on the diffusion of green technology innovation from the perspective of supply and demand. Dong et al. (44) analyzed the impact of factors such as digital integration and green knowledge collaboration ability on digital green technology innovation from a quantitative perspective. Yin et al. (45) used stochastic differential game to study the sharing of low-carbon technologies between dominant and inferior firms in collaborative innovation systems. Compared with the above literature, the novelty of this study is reflected in the following aspects: First, the research perspective is more novel, and the research perspective of this study focuses on the media attention. At present, most articles on enterprise green technology innovation ignore the influence of media coverage, especially the influence of media report accuracy rate on green technology innovation. Therefore, the research perspective of this study is innovative and groundbreaking. Second, the research method is more novel, and this study adopts the evolutionary game method. In contrast to the method of differential game, which emphasizes the impact of short-term decisions, evolutionary game is a method of studying dynamic games using evolutionary ideas, focusing more on the process and outcome of long-term evolution. Green technology innovation, as an ecosystem engineering involving multiple agents, has a relatively complex strategy evolution and is a strategic equilibrium achieved by the continuous evolution of enterprises. Therefore, the method of evolutionary game is more applicable. Third, the research level is more comprehensive and in-depth. Since both the government and the public have important influences on enterprises’ green technology innovation and cannot be ignored, this study discusses the mutual influences among the relevant interest subjects of the government, enterprises, and the public based on system thinking. At the same time, previous studies only abstractly analyzed the evolutionary equilibrium strategy of the system, but this study also takes heavy polluting enterprises as an example to conduct numerical simulation after the evolutionary equilibrium analysis, so as to further intuitively demonstrate the evolutionary trajectory of the system strategy.

### 6.2 Conclusion

Based on perspective of stakeholders and evolutionary game theory, this study constructs a tripartite evolutionary game model of enterprise–government–public under the new media environment to analyze the equilibrium stabilities of relevant subjects and the influence of media reports, as well as simulate the possible evolutionary stable strategies in the green technology innovation system. The results show that:

(1) As a new social supervision channel, media play an important role in the green technology innovation system. New media reports can effectively reduce the information asymmetry between enterprise and their stakeholders. At the same time, the reputation pressure brought by media reports could have a great impact on the decision-making of enterprise, the government, and the public.

(2) Whether enterprise, government, or the public, its strategy stability relies on the strategic choices of the other two subjects, and the accuracy of new media reports plays an important role in this process. This also reflects the necessity of studying the cooperation among enterprises, government, and the public in the new media environment for the formation of green technology innovation.

(3) Under the new media environment, enterprise–government–public tripartite game system may have four evolutionary stable strategies: (1,0,0), (0,0,1), (1,0,1), and (1,1,1). The evolution results not only depend on the internal benefits and costs of the three subjects but also are affected by the reputation of external new media reporting.

(4) The numerical simulation of the evolutionary stable strategy shows that the change of the initial probability value of each subject only affects the speed but not the result to the system evolution, and the authenticity of new media reports is an important factor to affect the evolution of green technology innovation system. A fair and just new media environment can effectively constrain enterprise traditional technology behavior, guide the public to actively participate in supervision, and play a certain role in replacing government regulation.

### 6.3 Managerial implications

Green technology innovation is very important as a strategic basis to solve the dilemma of green economy development and environmental protection. This study reveals the interactive process of green technology innovation-related subjects under the new media environment, which can provide some enlightenment for promoting green technology innovation of enterprises. How to exert the influence of the media’s public opinion to regulate the behavior of the government, enterprises, and the public will be an important breakthrough in promoting green technology innovation. First of all, the public and the new media, as “third party supervisors,” should establish an overall view of social governance, improve their sense of social responsibility, and consciously participate in the supervision of enterprise and government behavior. Second, the government should make great efforts to develop new media to make it become an effective supplement and support for government regulation; and establish a government-media information-sharing platform to realize information interaction and transparency among the public, the government, and new media. Finally, the new media itself should abide by professional ethics, take rigorous scientific attitude and knowledge as a basis of reporting, and make use of and rely on Internet technology to provide ways and means for public supervision in the process of enterprise green technology innovation, so as to further promote the realization of the dual goals of economy and ecology.

### 6.4 Practical and social implications

This study has important theoretical and practical significance. Based on the reality of China and the background of green sustainable development and the significant constraints of natural resources and environment faced by Chinese enterprises, this study deeply discusses the evolutionary balance strategy of green technology innovation system under the new media environment. This study not only provides a different perspective from the existing literature to analyze the formation of green technology innovation and enrich the relevant theories of media environmental governance but also provides ideas for manufacturing enterprises to adjust the decision-making behaviors related to green technology innovation under the new media environment. It is of great reference value to establish an environmental governance system jointly governed by the government, enterprises, the public, and the media.

### 6.5 Limitations

Although this study has achieved its intended objectives, deficiencies remain that are worthy of attention. This study only focuses on the impact of the correct rate of new media reports on the strategies of enterprise, the government, and the public. However, as a rational person who focuses on obtaining economic benefits, the new media may conspire with the government and enterprise, which is not taken into account in this study. Therefore, subsequent research will try to further explore other possible relationships among new media, the government, and enterprise, so as to provide more accurate reference for realizing the good evolution of green technology innovation system.

#### Remarks

① Replicator dynamic equation is the core concept of the evolutionary game, which is used to express the dynamic differential equation of the probability of an agent choosing a certain strategy in a group. The expression is as follows: f(α) = d(α)/d(t) = α*(U1-Uα).

② Evolutionarily stable strategy (ESS) is a kind of equilibrium strategy that the bounded rational subjects in the population constantly adjust their strategies according to their vested interests to seek improvement of their own interests and finally realize. This strategy brings more benefits to the population than other strategies.

## Data availability statement

The original contributions presented in the study are included in the article/supplementary material, further inquiries can be directed to the corresponding author/s.

## Author contributions

YL: thesis architecture design, writing the original draft. Y-pC: supervision and project administration. T-pX and Y-hX: reviewing, preparing final draft, and formatting. All authors read and approved the final manuscript.

## References

[1] Stucki T. What hampers green product innovation: the effect of experience. Ind Innov. (2019) 26:1242–70. doi: 10.1080/13662716.2019.1611417, PMID: 26811880

[2] Porter ME Linde CVD. Toward a new conception of the environment-competitiveness relationship. J Econ Perspect. (1995) 9:97–118. doi: 10.1257/jep.9.4.97

[3] Berrone P Fosfuri A Gelabert L Gomez-Mejia LR. Necessity as the mother of ‘green’ inventions: institutional pressures and environmental innovations. Strateg Manag J. (2013) 34:891–909. doi: 10.1002/smj.2041

[4] Horbach J Rammer C Rennings K. Determinants of eco-innovations by type of environmental impact—the role of regulatory push/pull, technology push and market pull. Ecol Econ. (2012) 78:112–22. doi: 10.1016/j.ecolecon.2012.04.005

[5] Chen YP Liu Y. The influence mechanism of media attention on green technology innovation of heavy polluting enterprises: based on the mediating effect of government environmental regulation and public participation. Manag Rev. (2023) 35:111–22. doi: 10.14120/j.cnki.cn11-5057/f.2023.06.020

[6] Harnesso L. Media technologies: Essays on communication, materiality, and society. Mobile Media & Communication. (2015) 3:286–7. doi: 10.1177/2050157914566911

[7] Li Y Wang L. Media supervision, reputation community and investor protection. Manage World. (2013) 11:130–143+188. doi: 10.19744/j.cnki.11-1235/f.2013.11.012

[8] Tang Z Tang J. Can the media discipline Chinese Firms' pollution Behaviors? The mediating effects of the public and government. J Manag. (2016) 42:1700–22. doi: 10.1177/0149206313515522

[9] Schiederig T Tietze F Herstatt C. Green innovation in technology and innovation management–an exploratory literature review. R&D Manag. (2012) 42:180–92. doi: 10.1111/j.1467-9310.2011.00672.x

[10] Wang MY Liu Y Yang WK. Study on the evolution game of regional cooperation in emission reduction under environmental regulation. China Manage Sci. (2019) 27:158–69. doi: 10.16381/j.cnki.issn1003-207x.2019.02.016

[11] Nie L Zhang LJ. Evolutionary game analysis and simulation of green technology innovation between government and polluters. Econ Issues. (2019) 10:79–86. doi: 10.16011/j.cnki.jjwt.2019.10.012

[12] Xu JZ Guan J Zhu XY. An evolutionary game study on the influence of government behavior on green innovation mode selection of manufacturing enterprises. Operat Res Manage. (2017) 26:68–77. doi: 10.12005/orms.2017.0212

[13] Ji GJ Liu H. Evolutionary game analysis of product ecological innovation for remanufacturing. Sci. Technol. Manag. (2013) 34:66–75.

[14] Li WH Li N Liu F. Three-group evolutionary game of green technology innovation stakeholders and its simulation. Operat Res Manage. (2021) 30:216–24. doi: 10.12005/orms.2021.0302

[15] Xu L Ma YG Wang XF. Environmental policy choice of green technology innovation based on evolutionary game: government behavior VS public participation. China Manage Sci. (2022) 30:30–42. doi: 10.16381/j.cnki.issn1003-207x.2020.1786

[16] Cao X Zhang LP. An evolutionary game analysis of Enterprise green technology innovation under environmental regulation: based on stakeholder perspective. Syst Eng. (2017) 35:103–8.

[17] Encarnacao S Santos FP Santos FC. Paths to the adoption of electric vehicles: an evolutionary game theoretical approach. Transport Res Part B Methodol. (2018) 113:24–33. doi: 10.1016/j.trb.2018.05.002

[18] Qu GH Liu X Qu WH Zhang Q. An evolutionary game analysis of the development strategies of government and game enterprises under public participation. Chin J Manage Sci. (2020) 28:207–19. doi: 10.16381/j.cnki.issn1003-207x.2019.0295

[19] Guan HL Wang Y Zhang H. An evolutionary game among government, enterprise and public: from the perspective of environmental regulation. Bus Res. (2022) 1:133–43.

[20] Heng C Yang Z Qi K. Research on the stability of green technology innovation Alliance for government-industry-university based on multi-party game. Operat Res Manage. (2021) 30:108–14. doi: 10.12005/orms.2021.0391

[21] Zheng MN Ren GQ. Evolutionary game analysis of enterprises’ green innovation behavior—based on the participation of environmental social organization. Operat Res Manage. (2021) 30:15–21. doi: 10.12005/orms.2021.0069

[22] Li CF Lu NN Li DD Wang XM. Enterprise green innovation: government regulation, information disclosure and investment strategy evolution. Stud Sci Sci. (2021) 39:180–92. doi: 10.16192/j.cnki.1003-2053.2021.01.013

[23] Tao XD. Giving full play to the role of modern media in environmental protection. News Front. (2017) 23:44–6.

[24] Ling S Li WM Liu XZ. Research on information game in emergent environmental events under new media context. J Inform. (2019) 38:149–57. doi: 10.3969/j.issn.1002-1965.2019.02.022

[25] Zhang KZ Shen JQ Xu SS Sun FH. Evolution game of government and Enterprise in Environmental Information disclosure: from the perspective of media supervision. Transac Beijing Institute Technol. (2019) 21:11–8.

[26] Ji XJ Chen HT Wang D. Media reports, government supervision and Enterprise environmental information disclosure. China Environ Manage. (2019) 11:44–54. doi: 10.16868/j.cnki.1674-6252.2019.02.044

[27] Xin Y. An empirical study on the impact of environmental regulation on green investment: from the perspective of media supervision. Friends Account. (2019) 14:91–6.

[28] Li Y. Public participation in the construction of social governance system under the background of new media. Fujian Forum. (2019) 5:156–64.

[29] Li D Zheng M Cao C Chen X Ren S Huang M. The impact of legitimacy pressure and corporate profitability on green innovation: evidence from China top 100. J Clean Prod. (2017) 141:41–9. doi: 10.1016/j.jclepro.2016.08.123

[30] Vaast E Safadi H Lapointe L Negoita B. Social media affordances for connective action: an examination of microblogging use during the Gulf of Mexico oil spill. MIS Q. (2017) 41:1179–205. doi: 10.25300/MISQ/2017/41.4.08

[31] Wang Y Li YX Ma Z Song JB. Media attention, environmental regulation and enterprise environmental investment. Nankai Manage Rev. (2017) 20:83–94.

[32] Agle BR Donaldson T Freeman RE. Dialogue: toward superior stakeholder theory. Bus Ethics Q. (2008) 18:153–90. doi: 10.5840/beq200818214

[33] Xu GW. The logic of das Kapital volume II: phylogenetics. Contemp Econ Resh. (2012) 1:1–7+92.

[34] Wang MY Li YM. Government market regulation drives Enterprise green technology innovation mechanism. China Sci Technol Forum. (2020) 6:85–93. doi: 10.13580/j.cnki.fstc.2020.06.016

[35] Xu JZ XC L. Research on the evolution of decision-making behavior of government, manufacturing enterprises and consumer groups under low-carbon economy. Operat Res Manage. (2014) 23:81–91.

[36] Wang W Zhang Z. Influence of innovation subsidy and failure compensation on green innovation strategy selection of enterprises. Soft Sci. (2019) 33:86–92. doi: 10.13956/j.ss.1001-8409.2019.02.18

[37] Freedman S Jin GZ. Learning by doing with asymmetric information: evidence from Prosper.com. Natl Bureau Econ Res. (2011):203–12. doi: 10.3386/w16855

[38] Long H Liu H Li X Chen L. An evolutionary game theory study for construction and demolition waste recycling considering green development performance under the Chinese Government’s reward–penalty mechanism. Int J Environ Res Public Health. (2020) 17:6303. doi: 10.3390/ijerph17176303, PMID: 32872529 PMC7503538

[39] Smith J Price GR. The logic of animal conflict. Nature. (1973) 246:15–8. doi: 10.1038/246015a0, PMID: 38136858

[40] Selten R. A note on evolutionarily stable strategies in asymmetric animal conflicts. J Theor Biol. (1980) 84:93–101. doi: 10.1016/S0022-5193(80)81038-1, PMID: 7412323

[41] Friedman D. Evolutionary games in economics. Econometrical. (1991) 59:637–66. doi: 10.2307/2938222, PMID: 38169894

[42] Lyapunov AM. The general problem of the stability of motion. Int J Control. (1992) 55:531–4. doi: 10.1080/00207179208934253, PMID: 38129244

[43] Wang M Lian S Yin S Dong H. A three-player game model for promoting the diffusion of green technology in manufacturing enterprises from the perspective of supply and demand. Mathematics. (2020) 8:1585–610. doi: 10.3390/math8091585

[44] Dong T Yin S Zhang N. The interaction mechanism and dynamic evolution of digital green innovation in the integrated green building supply chain. Systems. (2023) 11:122–46. doi: 10.3390/systems11030122

[45] Yin S Li B. A stochastic differential game of low carbon technology sharing in collaborative innovation system of superior enterprises and inferior enterprises under uncertain environment. Open Mathematics. (2018) 16:607–22. doi: 10.1515/math-2018-0056

